# Validation of the Modes of Transmission Model as a Tool to Prioritize HIV Prevention Targets: A Comparative Modelling Analysis

**DOI:** 10.1371/journal.pone.0101690

**Published:** 2014-07-09

**Authors:** Sharmistha Mishra, Michael Pickles, James F. Blanchard, Stephen Moses, Zara Shubber, Marie-Claude Boily

**Affiliations:** 1 Department of Infectious Disease Epidemiology, Imperial College, London, United Kingdom; 2 St. Michael's Hospital, Department of Medicine, University of Toronto, Toronto, Canada; 3 Centre for Global Public Health, Department of Community Health Sciences, University of Manitoba, Winnipeg, Canada; Alberta Provincial Laboratory for Public Health/University of Alberta, Canada

## Abstract

**Background:**

The static Modes of Transmission (MOT) model predicts the annual fraction of new HIV infections acquired across subgroups (MOT metric), and is used to focus HIV prevention. Using synthetic epidemics via a dynamical model, we assessed the validity of the MOT metric for identifying epidemic drivers (behaviours or subgroups that are sufficient and necessary for HIV to establish and persist), and the potential consequence of MOT-guided policies.

**Methods and Findings:**

To generate benchmark MOT metrics for comparison, we simulated three synthetic epidemics (concentrated, mixed, and generalized) with different epidemic drivers using a dynamical model of heterosexual HIV transmission. MOT metrics from generic and complex MOT models were compared against the benchmark, and to the contribution of epidemic drivers to overall HIV transmission (cumulative population attributable fraction over t years, PAF_t_). The complex MOT metric was similar to the benchmark, but the generic MOT underestimated the fraction of infections in epidemic drivers. The benchmark MOT metric identified epidemic drivers early in the epidemics. Over time, the MOT metric did not identify epidemic drivers. This was not due to simplified MOT models or biased parameters but occurred because the MOT metric (irrespective of the model used to generate it) underestimates the contribution of epidemic drivers to HIV transmission over time (PAF_5–30_). MOT-directed policies that fail to reach epidemic drivers could undermine long-term impact on HIV incidence, and achieve a similar impact as random allocation of additional resources.

**Conclusions:**

Irrespective of how it is obtained, the MOT metric is not a valid stand-alone tool to identify epidemic drivers, and has limited additional value in guiding the prioritization of HIV prevention targets. Policy-makers should use the MOT model judiciously, in combination with other approaches, to identify epidemic drivers.

## Introduction

Policy-makers routinely select and prioritize subgroups to target interventions with a goal to mitigate local HIV epidemics [1], [2]. Epidemic drivers are defined as subgroups or behaviors that are necessary and sufficient to enable HIV to establish and persist in a region [3]. Identifying those at highest risk of acquiring and transmitting HIV lets us design and focus interventions on behaviours that disproportionately sustain HIV spread [1], [4]. However, there are concerns that the most widely used tool to appraise HIV epidemics and help focus country-specific HIV prevention [5]–[8] – the UNAIDS “Modes of Transmission” (MOT) model [8], [9] - may not consistently identify epidemic drivers and therefore, may not adequately guide prevention[10].

The MOT is a static mathematical model that predicts the annual distribution of new HIV infections acquired by different risk-groups, herein referred to as the “MOT metric” [6], [8], [9] - a quantity often referred to as the current “source of HIV infections” [5], [11] and sometimes mistakenly interpreted to mean the highest-ranking subgroup “drives the epidemic” [7], [11], [12]. The MOT metric is used by policy-makers to inform prevention by identifying subgroups predicted to acquire the largest fraction of new HIV infections in the coming year. In many cases, the model predicts that most new HIV infections are acquired by low-activity groups in stable partnerships [13], [14], even in concentrated epidemics driven by commercial sex [10], [14]. This apparent contradiction raises three key concerns about the utility of the MOT model to identify epidemic drivers and to adequately inform prevention efforts [5], [10].

First, the structural simplicity of the generic MOT template has been criticized because it does not account for differential infectiousness by HIV stages, because heterogeneity in risk is limited to a few mutually exclusive subgroups (female sex workers [FSWs], clients, men who have sex with men, people who inject drugs, individuals with multiple partnerships, main or spousal partners of those engaged in high-risk behaviours, and the remaining low-activity population), and because individuals have only one type of HIV exposure (injecting drug use or sex) or partnership (commercial, casual, or spousal sex) [9]. Although the generic MOT can be modified to reflect the local setting in more detail if sufficient technical expertise and data is available [8], [14], none of the published MOT analyses have incorporated multiple HIV exposures, and only five have modified the generic MOT with additional subgroups [13]–[18]. An overly-simplified model structure may produce biased MOT estimates, and partly explain why the generic MOT model does not identify epidemic drivers [14].

Second, using biased or implausible input parameters may produce biased MOT metric estimates [5], [10], [19]. Because the MOT model is not calibrated to observed HIV prevalence or incidence, we cannot determine if our parameter combinations reliably reproduce the observed HIV epidemic trends. For example, input parameters such as population size and behavior of high-risk groups are often lacking or underestimated [10], [20], [21], which may partly explain why MOT models fail to identify epidemic drivers [10], [19].

Third, the MOT metric itself may be inadequate to identify the real epidemic drivers because it measures the short-term distribution of those who get infected, rather than those who transmit HIV infection. The MOT model does not capture the longer chains of secondary (indirect) transmissions due to high-risk behaviours [5], and does not account for the sexual life-course of individuals whose risk-taking behavior may change over time (in the absence of intervention) [10]. Hence, even reliable unbiased estimates of the MOT metric may underestimate the contribution of high-risk behaviors to overall HIV transmission, especially in the long-term as the number of secondary transmitted events increases over time [22]–[24].

Despite its extensive use in guiding policy[6], the MOT model has never been formally validated as a tool to prioritize HIV prevention efforts. Recent studies showed large differences in MOT metric estimates across data quality [10] and model complexity[14], but were unable to determine which estimates were closer to the truth because they did not use an independent reference benchmark [10], [14]. Therefore, we performed a comparative modelling analysis to objectively validate estimates of the MOT metric and the utility of the MOT model as a tool to identify epidemic drivers, and to select and prioritize HIV prevention targets as follows. We developed a complex dynamical mathematical model to simulate synthetic data from three epidemiologic contexts (concentrated, mixed, and generalized HIV epidemics). We used the synthetic data to generate our benchmark MOT metrics and to identify the epidemic drivers in each epidemic type. The MOT model was then applied to the same synthetic data to derive different estimates of the MOT metric using increasingly complex MOT models. In the absence of an empirical gold standard, using synthetic data is the best and only option to objectively compare the MOT metric against a known benchmark, and answer the following questions: (1) are structural simplicity, biased parameters, or the use of a static model leading to unreliable MOT metrics which underestimate the importance of epidemic drivers, or is it the MOT metric itself that limits the validity of the MOT model in identifying epidemic drivers; (2) even if we could estimate the unbiased MOT metric, is this information useful for focusing HIV prevention?

## Methods

**Figure 1:**
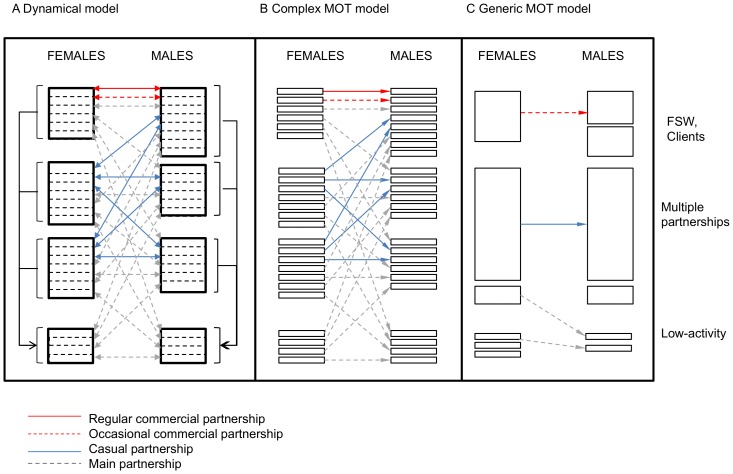
Sexual structure of the dynamic model, complex MOT model, and generic MOT model. (A) In the dynamical model, the population is divided into four different activity classes based on the frequency of yearly partner change (FSWs/clients, two multiple partnership classes, and a low-activity class). Four partnership types are possible: commercial (regular or occasional), casual, or main. In the dynamical model, males and females who engage in higher-risk activity (commercial or casual sex) cease higher-risk activity and enter into the low-activity population reflecting a turn-over in each of the higher-risk activity groups (solid black lines). Multiple concurrent exposures are possible, and subpopulations are linked via bridging groups (individuals with multiple exposures). The partnerships are therefore shown with double-headed arrows to represent bridging between groups. (B) The complex Modes of Transmission model (cMOT) divides the population into the same activity classes as the dynamical model. The cMOT allows for multiple exposures to HIV (i.e. multiple types of partnerships). For visibility, only partnerships where infections are acquired by males are shown. Infections acquired by males and by females are counted separately, and partnerships are therefore shown with single-arrows to represent the lack of bridging between groups. Secondary infections and movement between risk-groups are not possible. (C) The generic Modes of Transmission model (gMOT) uses a simplified sexual structure, and only partnerships where infections are acquired by males are shown. In the gMOT, only one type of HIV exposure or partnership is possible, and subgroups are amalgamated in keeping with the generic MOT template [8], [9]. Infections acquired by males and by females are counted separately. As with the cMOT, single-headed arrows are used to represent different partnerships without bridging between groups. Hence, indirect transmission via bridging populations and secondary infections, and movement between risk-groups are not possible. MOT (modes of transmission); FSWs (female sex workers).

We developed four mathematical models. To generate the synthetic data, we developed a dynamical model of heterosexual HIV transmission with a relatively complex sexual structure and four HIV stages reflecting variation in CD4 level and HIV infectivity (4-stage dynamical model, Figure 1a, Figure S1). We then developed three models to generate the comparator MOT metrics: a 1-stage dynamical model with uniform HIV infectivity that was otherwise exactly the same as the 4-stage dynamical model; and a pair of static MOT models (a complex MOT [cMOT, Figure 1b] and the generic MOT [gMOT, Figure 1c]). The synthetic data generated by the 4-stage dynamical model was used to parameterize the other three models (1-stage dynamical, cMOT, and gMOT). For example, the HIV prevalence inputs for the cMOT and gMOT were the prevalence outputs from the 4-stage dynamical model at the start of each year. Key differences between models are outlined in Table 1. See Text S1 for model equations and parameters.

**Table 1:** Differences between the models.

	4-stage DM (synthetic epidemics)	1-stage DM	Complex MOT (cMOT)	Generic MOT (gMOT)
**Input parameters**	Calibrated	Same as 4-stage DM, except for HIV infectivity	Same as 1-stage DM	Same as 1-stage DM
**Biological structure**				
Differential HIV infectivity by stage of HIV	Yes	No	No	No
STI co-factor increases HIV susceptibility per sex-act	Yes	Yes	Yes	Yes
STI co-factor increases HIV infectivity per sex-act	Yes	Yes	Yes	No
Condom-use reduces HIV transmission	Fraction of partnerships where condoms are used	Same as 4-stage DM	Same as 4-stage DM	Fraction of sex acts protected
**Sexual structure**				
Multiple HIV exposures	Yes	Yes	Yes	No
Turn-over between risk-groups	Yes	Yes	No	No
Subgroup size	See Table 2	Same as 4-stage DM	Same as 4-stage DM	Aggregate the two MP classes
Subgroup sexual behaviours	See Table 2	Same as 4-stage DM	Same as 4-stage DM	Weighted average of the two types of commercial partnerships, and two MP groups
Secondary, or indirect transmission events	Yes	Yes	No	No
**Can the model provide the following information?**				
The distribution of new HIV infections acquired by different subgroups (MOT metric)?	Yes	Yes	Yes	Yes
What is the fraction of new HIV infections transmitted from a given subgroup?	Yes	Yes	Yes	Yes
Estimate contribution of specific partnerships/risk-groups to overall transmission in the total population, over t years?	PAF_t_	PAF_t_	No	No

DM (dynamical model). MOT (Modes of Transmission model). PAF_t_ (population attributable fraction over t years) reflects the contribution of each type of partnership to overall transmission. MP (multiple partnership). STI (sexually transmitted infection; in this study, only HSV-2 is considered).

### Dynamical models

The compartmental, deterministic model divides the synthetic population into four activity classes, including FSWs and their male clients (Figure 1, Table 2). Movement from high- to low-activity classes were included, reflecting, for example, the retirement of FSWs (Figure 1, Text S1). Individuals formed at least one of four types of sexual partnerships (Figure 1). The model included baseline male circumcision (reduced HIV susceptibility in males), and co-factor effects of a concomitant sexually transmitted infection (herpes simplex virus type 2).

**Table 2:** Epidemiologic context and parameters.

	Concentrated	Mixed	Generalized
Epidemiologic features			
Setting used to generate synthetic epidemics	Belgaum, India	Kisumu, Kenya	Lesotho
Epidemic drivers	Commercial sex	Commercial sex and multiple partnerships	Multiple partnerships
Level of condom-use among epidemic drivers	High (≥75%) [35], [36]	Medium (30–58%) [37]	Low (40%) a
Population size			
Clients (% of adult males in 2005)	17.0 [10]	8.7 [38]	1.9[39]
Total MP males (High-frequency, Intermediate-frequency; % of adult males in 2005)	9 (2,7)^a^ [20], [40], [41]	23 (7,15)^a^ [38]	23(3,21) a [39], [42]
FSWs (% of adult females in 2005)	0.8[41]	3.0 [43], [44]	0.8[45]
Total MP females(High-frequency, Intermediate-frequency; % of adult females in 2005)	5(1,4)^a^ [20], [40], [41]	23 (7,15)^a^ [38]	23(2,21)^a^ [39], [42]
Duration of time spent in each higher-activity class (years)			
FSW	8 [46]	1 [47]	1^a^
Clients	20 [48]	10	10
MP (assumption) b	10	10	10
Fraction who form casual partnerships (%)			
FSWs, and currently low-activity	0	0	0
Clients	10^a^	43 [49]	40^a^
MP	100	100	100
Fraction with a main partnership (%)			
FSWs	50 [46]	80 [47], [50]	70^a^ ^c^
Clients	65[48]	72 [49]	70^a^ ^c^
MP	65[40]	50 [38]	70^a^ ^c^
Currently low-activity	100	100	100
Partner frequency by partnership type (per year)			
FSWs (occ. commercial, reg. commercial, main)	500 [46], 40[46], 1	104 [47], [50], 7.8^a^, 1	40, 1^a^, 1
Clients (occ. commercial, reg. commercial, casual, main)	24 [48], 2.4, 3, 1	36^a^, 3.6^a^, 5, 1	2 a, 0.8^a^, 20, 1
High-frequency MP (casual, main) d	3 [41], 1	6^a^, 1	20^a^, 1
Intermediate-frequency MP (casual, main) d	2 [41], 1	2^a^, 1	3 a, 1
Currently low-activity (main)	1	1	1
Number of sex acts per year and consistent baseline condom-use (%) within each partnership type			
Main	104 [40], 10% [40]	104^a^, 10% [51]	124^a^, 10% [39]
Casual	12^a^, 40%^a^ [40]	48^a^, 30% [51]	52^a^, 40% [39]
Regular commercial	18^a^, 75% [46]	14^a^, 55% [50]–[54]	12^a^, 40% [39]
Occasional commercial	1, 85% [46]	1, 58% [51]–[54]	1, 58% [45]
Proportion of clients that form regular commercial partnerships with FSWs (%)	40 [46]	80^a^	5^a^
Prevalence of sexually transmitted infection (HSV-2) (%)			
Clients, ex-clients	60.0 [41], [48]	58.0 [54], [55]_ENREF_40	50.0 [55], [56]
MP, ex-MP males	18.0 [40]	58.0 [53], [54]	50.0 [55], [56]
Always low-activity males	13.0 [40]	34.0 [54]	34.0 [55], [56]
FSW, ex-FSW	80.0 [41], [46]	94.0 [47]	65.0 [55], [56]
MP, ex-MP females	18.0 [40]	67.7 [54]	65.0 [55], [56]
Always low-activity females	13.0 [40]	67. 7 [54]	65.0 [55], [56]

a Calibrated estimate.

b The duration of high-risk sex in the MP class was assumed to be approximately one-third of the total duration of sexual activity. The total duration of sexual activity was assumed to be 34 years in all regions.

c Lesotho: range (45–80) used for model calibration and set to equal levels across higher-risk groups to minimize the number of parameters to fit.

d When calibrating for the two MP groups, the weighted average for partner frequency was restricted to 2–3 (Belgaum), or 2–8 (Kisumu/Lesotho) [39], [42], [57].

MP (multiple partnership) groups. FSW (female sex workers).HSV-2 (herpes simplex virus type 2).

### MOT models

The cMOT matches the sexual structure of the dynamical model allowing for multiple HIV exposures and partnerships (Figure 1b, Table 1). In keeping with the generic template, our gMOT merges subgroups and partnerships, and does not allow for multiple HIV exposures (Figure 1) [8], [9]. Neither MOT model includes secondary transmission or movement between activity classes.

### Epidemic types

We used region-specific data from south India (Belgaum), Kenya (Kisumu), and Lesotho (country-level data) to derive plausible parameter values and simulate our synthetic epidemic with the 4-stage dynamical model (Figures S2, S3, and S4). These regions were chosen for their different epidemiologic contexts (overall HIV prevalence and characteristics of commercial sex), and the geographic-level (national or sub-national) reflects the geographic-scope of available data. We then classified each synthetic epidemic as being concentrated, mixed, and generalized [1], [4], [10], [23], [25]. The concentrated epidemic required that commercial sex exist for HIV to establish and persist in the population, meaning that the basic reproductive ratio (average number of new infections due to one infectious case in an otherwise susceptible population, R_0_) is greater than 1 in the presence of commercial sex and <1 in the absence of commercial sex. The generalized epidemic required multiple partnerships (casual sex) for R_0_ to be greater than 1, such that existing commercial sex was neither sufficient nor necessary for R_0_ to exceed 1. The mixed epidemic required either commercial sex or casual sex for HIV to establish and persist, such that both commercial and casual sex acts would need to be protected to achieve long-term elimination (R_0_ <1). Each synthetic epidemic was classified by examining the counterfactual (‘turning off’ transmission within specific partnerships, Figure S5). Key differences between the synthetic epidemics are listed in Table 2.

### Calculation of the MOT metric

We used the synthetic data on the annual number of new HIV infections by risk groups to derive the benchmark MOT metric. We then parameterized the 1-stage dynamical model and both MOT models with the same synthetic data to derive the MOT metric from each comparator model. The inputs for the gMOT represent a weighted average of the parameters for the relevant subgroups that were merged as per the generic MOT template[9]. Each comparator model assumes constant HIV infectivity throughout infection, and uses a weighted average of the transmission probability from the 4-stage dynamical model.

The MOT metric is reported in line with the policy literature [7], by summing the number of HIV infections acquired by FSWs, clients, or individuals in the high- and intermediate-frequency multiple partner (MP) class. The remainder of infections occur in the low-activity class and are divided among those acquired by main or spousal partners of clients, main or spousal partners of the MP class, and among individuals where both partners reside in the low-activity class.

### Validity analysis of the MOT metric

We assessed the validity of the MOT metric in the following four stages:

1. To assess the sensitivity of the MOT metric to biological and sexual structure (structural uncertainty), and model type (static vs. dynamic), we compared the MOT metrics obtained from our three comparator models to the benchmark MOT metric.
2. To assess the sensitivity of the MOT metric to potentially biased parameters, we compared the MOT metric from the cMOT using unbiased input parameters (i.e. parameters from the synthetic epidemics), to the MOT metric using biased parameters, which were varied one by one. In keeping with social desirability bias observed in behavioural surveys [20], [21], the size of high-risk subgroups was varied by 0 to 100%. All other (47) parameters were varied by ± 50%.
3. To assess the validity of the MOT metric itself in identifying the relevant prevention targets over time, we compared the benchmark MOT metric at different time-points with the known epidemic drivers.
4. To assess the extent to which the MOT metrics (including the benchmark MOT metric) reflect the short- and long-term contribution of different subgroups to overall HIV transmission, we compared the MOT metrics with the cumulative population attributable fraction of infections (PAF_t_) from corresponding partnerships over one year (PAF_1_) and over 5–30 years (PAF_5_ - PAF_ 30_), starting in 2012. The PAF_t_ is estimated using the 4-stage dynamical model by “turning off” HIV transmission from specific subgroups in 2012, and calculating the relative difference in the number of new HIV infections acquired by the total population over time (*t* years). We then compared the different MOT metrics (reflecting HIV acquisition) and the PAF_t_ with the one-year fraction of HIV infections transmitted from the corresponding subgroups using the cMOT (“cMOT transmitted”). Details are provided in Text S1.

### Prevention implications of using the MOT metric to guide policies

We used the 4-stage dynamical model to assess the potential impact on HIV incidence of introducing HIV prevention policies guided by the gMOT metric in 2012. For the illustrative purpose of this analysis, we applied a generic intervention that reduces per-act transmission by 80% under a resource cap specific to each setting. The number of person-years of intervention was capped at the number required to increase condom-use within commercial partnerships to 98% (assuming both FSWs and clients agree to use condoms) in the concentrated epidemic; the number required to double condom-use within casual partnerships in the generalized epidemic; and number required to increase condom-use within commercial partnerships to 98% and double condom-use within casual partnerships in the mixed epidemic.

Four policies were examined (Table 3). Policies 1 and 2 used the ranking from the gMOT (as is most commonly cited in the policy literature [7], [11], [12], [26]) to prioritize interventions to the subgroup with the largest burden of new infections. Policy 1 re-distributed resources by reducing the coverage of existing condom-based intervention programs for FSWs and/or the MP groups and re-allocated these resources to the subgroup with the largest burden of new HIV infections. Policies 2 to 4 assumed that resources were added to existing interventions, so condom-use was maintained at 2012 levels. Policy 3 used additional resources to target intervention to epidemic drivers (based on an increasing long-term PAF_t_). Policy 4 randomly distributed additional resources across subgroups, mimicking a situation without an epidemic appraisal. Each policy was implemented in 2012, with immediate scale-up and sustained efforts.

**Table 3:** Prevention policies under finite resources, as formulated in 2012.

Policy description	Resources for existing interventions	Population	Intervention
1. gMOT-directed: resources are prioritized to subgroup with the largest burden of new HIV infections in 2012	Redistributed a	Low-activity	generic
		Multiple partners	↓condoms
		FSWs and clients (occasional)	↓condoms
		FSWs and clients (regular)	↓condoms
2. gMOT-directed: additional resources prioritized to reach the subgroup with the largest burden of new HIV infections	Continued	Low-activity	generic
		Multiple partners	↔condoms
		FSWs and clients (occasional)	↔condoms
		FSWs and clients (regular)	↔condoms
3. Directed by Increasing long-term PAF_t_: additional resources prioritized to epidemic drivers b	Continued	Low-activity	generic
		Multiple partners	↔condoms + generic
		FSWs and clients (occasional)	↔condoms + generic
		FSWs and clients (regular)	↔condoms + generic
4. No epidemic appraisal: random distribution of additional resources	Continued	Low-activity	generic
		Multiple partners	↔condoms + generic
		FSWs and clients (occasional)	↔condoms + generic
		FSWs and clients (regular)	↔condoms + generic

MOT (modes of transmission).

a Under policy 1, resources were redistributed from high-risk groups (condom-use coverage was reduced). Initiation of combination antiretroviral treatment is assumed to be equal across subgroups, and not affected by the prevention policies examined here.

b Concentrated epidemic (FSWs and clients; commercial sex), Generalized epidemic (individuals with multiple partnerships; casual sex), Mixed epidemic (FSWs, clients, and individuals with multiple partnerships; commercial and casual sex)

↔ (levels remained stable after 2012); ↓ (coverage declined after 2012).

## Results

The epidemic driver in the concentrated, mixed, and generalized epidemic was commercial sex, a combination of commercial and casual sex, and casual sex, respectively (Table 1, Figure S5).

### Sensitivity of the MOT metric estimates to biological, sexual, and static structure

**Figure 2:**
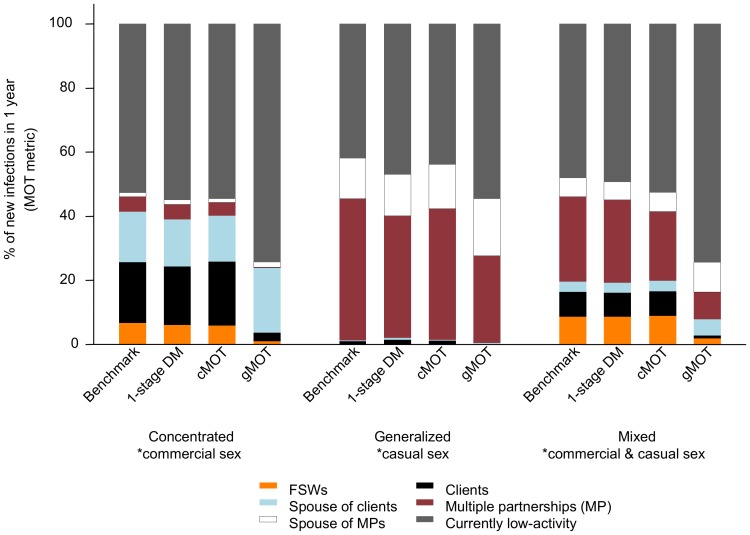
Model-predicted distribution of new HIV infections over one year (MOT metric) in three epidemic types. The benchmark Modes of Transmission (MOT) metric is obtained from the 4-stage dynamical model, and corresponding MOT metric obtained from the 1-stage dynamical model (1-stage DM), complex MOT (cMOT), and generic MOT (gMOT) models. The MOT metric reflects the fraction of new HIV infections acquired by different risk groups (colored bars) estimated for 2012 using data from the synthetic epidemics.*local epidemic drivers.

Figure 2 compares the 2012 MOT metrics from different models with the benchmark. The MOT metrics generated by the 1-stage dynamical model and the cMOT were similar to the benchmark. This suggests that assumptions of uniform HIV infectivity in the MOT models do not substantially bias the MOT metric estimates. In contrast, the MOT metric was very sensitive to assumptions about sexual structure. Using the gMOT biased the predicted distribution of new HIV infections toward the low-activity group in all epidemics (Figure 2, Figures S6–S8). Across all three epidemic types, all models including the benchmark MOT predicted that most new HIV infections were acquired by low-activity groups in 2012 (Figure 2) rather than among local epidemic drivers. Hence, using a simplified sexual structure in the MOT model reduces the validity of the MOT metric estimate, but does not explain why MOT models do not identify local epidemic drivers.

### Sensitivity of the MOT metric estimates to biased parameter inputs

**Figure 3:**
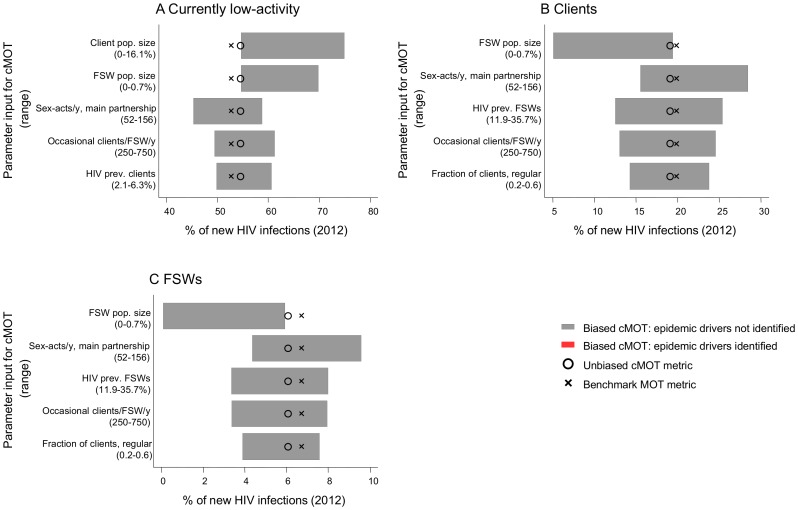
Sensitivity of the MOT metric to biased input parameters (concentrated epidemic). The range in the predicted fraction of new HIV infections acquired by the low-activity group (A), clients (B), and female sex workers (FSWs, C) are depicted for the five most influential parameters from the complex Modes of Transmission model (cMOT) using biased inputs. Also shown are the benchmark MOT metric and the unbiased cMOT metric. Across the parameter range examined here, the low-activity group incurred the largest burden of new infections, and the unbiased MOT metric did not identify the epidemic driver (no red regions). Pop. (population); Prev. (prevalence).

Of the 51 parameters explored in univariate sensitivity analyses, the size, the HIV prevalence, and the frequency of partner change in high-risk groups, and the number of sex-acts within partnerships, were the most influential parameters on the cMOT metric across epidemic types (Figure 3, Figure S9, Figure S10). As expected, underestimates of the population size of high-risk groups led the cMOT metric to overestimate the relative burden of new HIV infections in the low-activity group (Figure 3, Figure S9, Figure S10). Overestimating sex acts within casual partnerships, or underestimating sex acts in main partnerships, led the biased cMOT to overestimate the burden of new infections in the multiple partnership groups and helped identify the local epidemic drivers; however, this effect was only observed in the generalized epidemic (Figure S9). The findings suggest that while biased inputs produce biased MOT metrics, and therefore, reduce validity, they do not explain why MOT models do not consistently identify local epidemic drivers.

### Validity of the MOT metric itself in identifying the relevant prevention targets (epidemic drivers) over time

**Figure 4:**
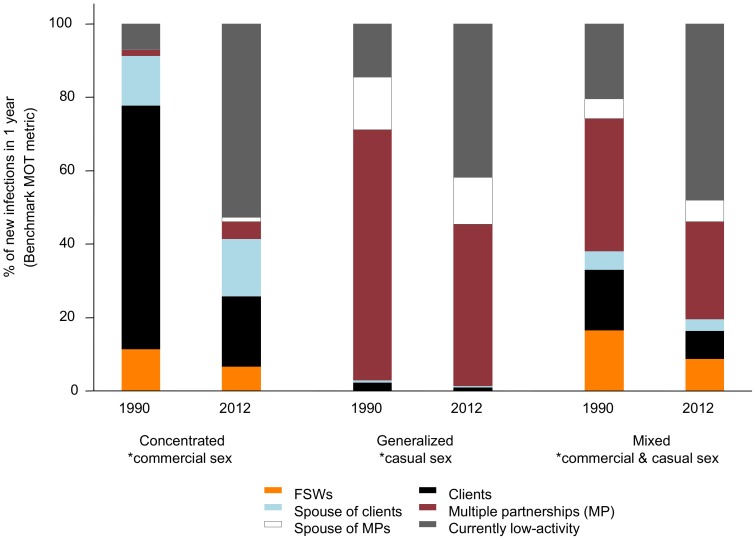
The benchmark MOT metric over time in three epidemic types. The benchmark Modes of Transmission (MOT) metric is obtained from the 4-stage dynamical model, and is shown for the years 1990 and 2012. The MOT metric reflects the fraction of new HIV infections acquired by different risk groups (colored bars) estimated for 2012 using data from the synthetic epidemics.*local epidemic drivers.

Figure 4 and Figures S6-S8 demonstrate how the benchmark MOT metric changes over the course of an epidemic. In the concentrated epidemic, most new infections (77%) were acquired by clients and FSWs in 1990, compared to the low-activity group in 2012 (65%). In the generalized epidemic, 67% of new infections occurred in the multiple-partnership group in 1990, compared to approximately 40% in each of the low-activity and multiple-partnership groups in 2012. Early in the mixed epidemic, most new infections occurred in FSWs and clients (37% in 1990), and the multiple-partnership group (39% in 1990), compared to the low-activity group in 2012 (48%). Hence, the benchmark MOT metric identified epidemic drivers early in the epidemics, but failed to do so in the later stages (with the possible exception of the generalized epidemic). Thus, even if perfectly estimated (as in the case of the benchmark MOT metric for our synthetic epidemics), the fraction of new HIV infections measured in a mature epidemic alone may not distinguish epidemic types, particularly between the concentrated and mixed epidemics (Figure 4).

### MOT metric and the contribution of epidemic drivers to overall HIV transmission

**Figure 5:**
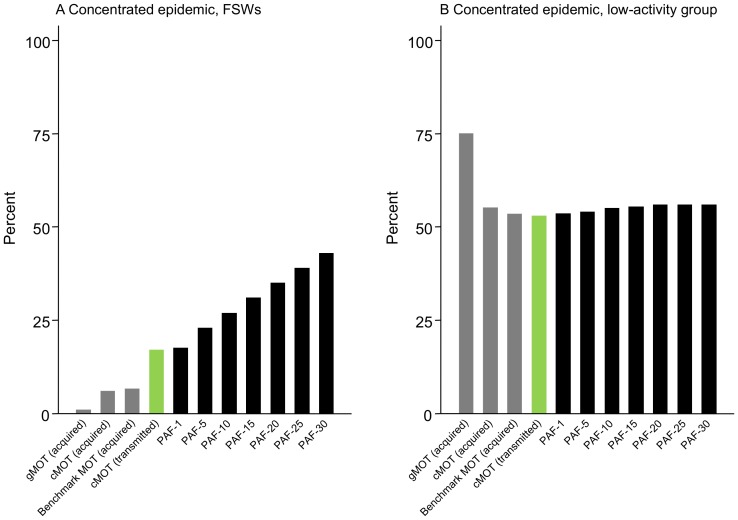
MOT metrics by subgroups and their contribution to overall HIV transmission (concentrated epidemic). The predicted fraction of new infections acquired by female sex workers (A, FSWs) and the low-activity group (B), as obtained from the complex Modes of Transmission model (cMOT acquired) and the generic Modes of Transmission model (gMOT acquired), and the benchmark MOT (acquired), are shown in grey. The fraction of HIV infections transmitted from FSWs and the low-activity group is shown in green (cMOT transmitted). The cumulative population attributable fraction (PAF_t_) over different time horizons measured from the year of the MOT (2012) for the epidemic driver (FSWs) and low-activity groups are shown in black.

The fraction of HIV infections acquired in different subgroups (MOT metrics), the fraction of HIV infections transmitted from each subgroup (“cMOT transmitted”), and the contribution of that subgroup to overall HIV transmission (PAF_t_) is depicted across epidemic types (Figure 5, Figure S11, Figure S12). In the concentrated and mixed epidemics (Figure 5a, Figure S12a), the fraction of HIV infections acquired by FSWs (benchmark MOT metric) underestimated the PAF_1_ of FSWs, while the fraction of HIV infections transmitted from FSWs (“cMOT transmitted”) approximated the PAF_1_ of FSWs. This is because HIV acquisition among FSWs (MOT metric) does not reflect HIV transmission to clients and other male partners over one year. In contrast, the benchmark MOT metric for the multiple-partnership and low-activity groups includes HIV infections acquired and transmitted between males and females within each respective group, and is thus similar to the cMOT metric and PAF_1_ (Figure S11a–b, Figure 5b, Figure 12b).

The cumulative PAF_t_ of the epidemic drivers increases over time (Figure 5a, Figures S11a–S12a). For example, over 30 years, FSWs contributed to 42%–47% of overall transmission in the concentrated and mixed epidemics, compared to 17–22% over one year (Figure 5a, Figure S12a). In the generalized epidemic, sex within multiple partnerships contributed to 64% of all transmission over 30 years (Figure S11a). In contrast, the cumulative PAF_t_ of sex within low-activity partnerships remained relatively stable over time (Figure 5b, Figure S11b, Figure S12b).

Therefore, even unbiased estimates of the MOT metric (infections acquired), unbiased estimates of the annual fraction of HIV infections transmitted from epidemic drivers, or unbiased estimates of the PAF_1_ would underestimate the medium- to long-term contribution of epidemic drivers to overall HIV transmission. This underestimate was largest with the gMOT (Figure 5, Figure S11, Figure S12). The findings were similar over the range of values explored in the univariate sensitivity analysis of biased inputs for the cMOT (data not shown).

### Prevention implications

**Figure 6:**
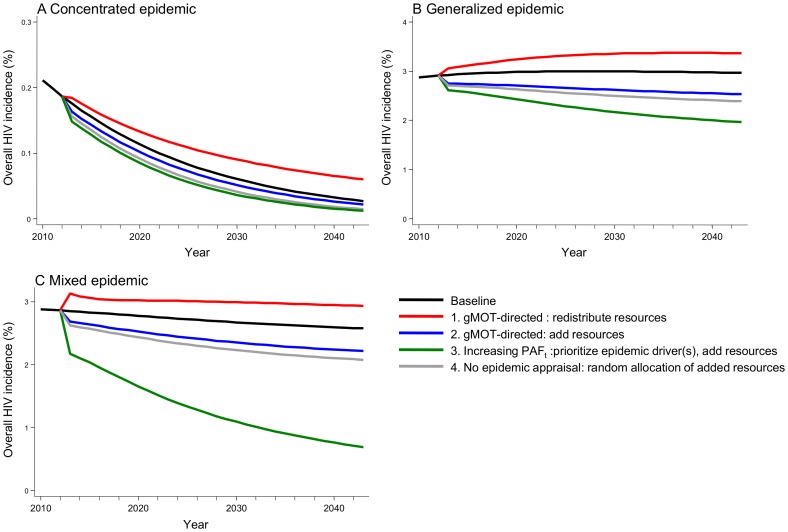
Impact of different prevention policies on the overall HIV incidence in three epidemic types. A generic intervention that reduces HIV transmission by 80% per sex-act is used. Policy 1 (red) prioritizes the low-activity group based on the largest burden of new HIV infections estimated from the generic Modes of Transmission model (gMOT) in 2012. gMOT-guided Policy 1 redistributes finite resources from condom-use coverage in high-risk groups to a generic intervention focus on low-activity individuals. gMOT-guided Policy 2 (blue) prioritizes the low-activity group but resources are added to existing interventions (baseline condom use in high-risk partnerships is sustained). Policy 3 (green) is guided by an increasing long-term population attributable fraction over time t (PAF_t_), and therefore prioritizes epidemic drivers to receive the generic intervention. Policy 4 is not informed by an epidemic appraisal, and randomly allocates additional resources across subgroups. Each policy is implemented in 2012, is immediately scaled-up, and sustained over 30 years of follow-up. The person-years of the generic intervention are fixed throughout the follow-up period, and equivalent within each simulated synthetic epidemic type.

Figure 6 illustrates the plausible consequences of prevention policies based on different epidemic appraisals (gMOT, long-term PAF_t_, none) when applied under an equivalent resource cap across epidemic types. Based on the gMOT, diverting resources away from epidemic drivers (Policy 1) fared the worst, leading to increased overall HIV incidence, while Policy 3 (prioritizing epidemic drivers based on the long-term PAF_t_) achieved the largest long-term impact. If additional resources were available and were focused on the low-activity population because the largest fraction of annual HIV infections were acquired within this group (gMOT-directed Policy 2), the result was a modest reduction in overall HIV incidence. Of note, random allocation of additional resources (Policy 4) – based on policies that did not use an epidemic appraisal and did not prioritize subgroups – achieved a similar or larger impact than MOT-guided policy 2.

## Discussion

### Validity of the MOT model and MOT metric

Epidemic tools used to guide HIV prevention policies aim to ensure that the right populations are reached, the most effective interventions are applied at scale, and finite resources are aligned with the desired goals for epidemic control [1], [27], [28]. Using a dynamical transmission model and synthetic data to objectively validate the MOT metric in a controlled and simulated environment, we demonstrated three key results.

First, the MOT metric was sensitive to simplifications in the sexual structure and biases in the input parameters leading to biased estimates of the annual distribution of new HIV infections obtained from MOT models. However, estimates were improved, and essentially, unbiased, when the structure of the MOT model was equivalent to that of our synthetic population and used unbiased parameters. This means that with better (less biased) data and a more detailed and tailored model structure, more reliable estimates of the MOT metric could be obtained [10], [14].

Second, we demonstrated that it was not the static MOT model per se, but the MOT metric itself – even if perfectly estimated or generated by a dynamical model - that was inadequate to identify the relevant prevention targets because it consistently underestimated the long-term contribution of epidemic drivers to overall HIV transmission (Table 4). Similarly, none of the other ‘short-term’ measures (the unbiased annual fraction of HIV infections transmitted, the annual PAF) captured the long-term contribution of epidemic drivers. Hence, improving the reliability of MOT model predictions by improving model structure or using better parameters is unlikely to be sufficient if our objective is to focus country-specific HIV prevention and achieve a long-term impact.

**Table 4:** Key Findings.

**The annual distribution of new HIV infections (MOT metric) is an inadequate metric, in and of itself, for identifying local epidemic drivers and prioritizing the relevant HIV prevention targets,** especially in mature HIV epidemics.
**An unbiased MOT metric does not consistently identify epidemic drivers because the metric inherently underestimates the long-term contribution of epidemic drivers to overall HIV transmission.** The contribution of epidemic drivers to overall transmission increases over time due to secondary transmitted events.
**Estimates of the MOT metric is sensitive to structural and parameter uncertainty, which exacerbate the bias in the annual fraction of HIV infections acquired by epidemic drivers, and the fraction of HIV infections due to epidemic drivers in the long-term.** Improving the MOT model (by improving sexual structure and parameterization) will improve the reliability of the MOT model's predictions.
**The validity of the MOT metric in identifying epidemic drivers cannot be improved** by increasing the complexity of the MOT model structure calibrating the MOT model (using ‘unbiased inputs’), or using a dynamical model (instead of a static model) to generate the metric.
**MOT-directed policies which do not prioritize HIV epidemic drivers could undermine our ability to reduce HIV incidence in the long-term.** MOT-directed HIV prevention policies are only useful in the short-term if existing interventions for epidemic drivers are sustained. MOT-directed policies may not perform better than random allocation of additional resources.

Third, translation of the MOT metric based on how subgroups rank by burden of newly acquired infections tended to prioritize the low-activity population across epidemic types. Thus, MOT-based policies often missed the role of epidemic drivers to overall HIV transmission, which undermined our ability to control our synthetic HIV epidemics in the long-term.

The failure of the MOT metric to identify epidemic drivers were exacerbated in the late (mature) epidemic phase, by simplifying the sexual structure of the MOT model, removing multiple HIV exposures, and when underestimating key parameters such as the size of high-risk groups. The benchmark MOT metric identified epidemic drivers in the early epidemic phase, but not later (mature) phases. In our synthetic generalized epidemic, the benchmark MOT identified the single epidemic driver (casual sex within multiple partnerships) even in the mature phase partly because individuals engaged in multiple partnerships were the largest risk group. As in most regions, the low-risk group in our synthetic epidemics was the largest risk group, which means that this group could acquire the largest burden of new HIV infections even if their per-capita HIV incidence rate is low [8]. This is evident from most published MOT analyses which identify the low-activity population as the most vulnerable subgroup, particularly if condom use is high within epidemic drivers (such as FSWs and clients) [6], [13].

### Prevention implications

The illustrative analysis of the impact of directly translating the MOT metric into policies prioritizing the low-activity group depended on how resources were allocated. MOT-guided policies did not perform better than random allocation of additional resources, highlighting the potential limited value in using the MOT to appraise epidemics and guide the selection of prevention targets. In addition, the prevention gains already made could even be reversed if resources are re-distributed based on the MOT results. The allocation of resources was illustrative and cannot be extrapolated to complex real-life choices. Nonetheless, it provides a simple exploration of what it could mean for HIV policies guided by expected short-term versus the longer-term impact.

In practice, HIV prevention targets based on the MOT vary across countries [2], [13], [29]. While some countries explicitly target interventions to the highest-risk groups and local epidemic drivers [4], many countries prioritize prevention to the “general population” (efforts which may or may not also address epidemic drivers) [13], [29]. The importance of prioritizing prevention to key populations, such as FSWs, in high-prevalence HIV epidemics has re-emerged in the policy discourse [30]-[32]. However, in regions such as sub-Saharan Africa, prevention efforts focused on key populations remain scarce, perhaps partly because the long-term contribution of key populations to overall HIV transmission and the potential long-term prevention benefits of key population interventions have been overlooked [29], [33]. Relying on the MOT metric, or any short-term metric, to characterize local HIV epidemics and prioritize HIV prevention targets could continue to misdirect resources away from epidemic drivers [7], [11], [12], [29].

### Strength and limitations

This is the first study to objectively assess the validity of the static MOT model [9] using synthetic epidemics generated by a dynamical HIV transmission model, and therefore, the first to evaluate the MOT model using an objective, benchmark MOT metric and epidemic drivers. The simulated settings were informed by regional empirical data in order to use realistic parameter values. The examination of three epidemic types improves generalizability of findings across epidemiologic context. This study also demonstrates the use of dynamical transmission models to simulate synthetic data, and how we can use them to test the validity and utility of different epidemic tools.

We did not examine other key populations, including men who have sex with men and people who inject drugs. However, the overarching principles with respect to the MOT metric are expected to be similar if the sexual partnerships between other high-risk groups and the rest of the population resemble the connectivity between risk-groups in the heterosexual HIV epidemics studied here. Although our deterministic model to generate the synthetic data was somewhat limited in structural complexity, its sexual structure included the important features of most published models that include high-risk groups[34], and exceeded that of the generic MOT model[9], [14]. A more complex model (e.g. with partnership duration) would have only increased differences between estimates of the static gMOT and the benchmark MOT, which would strengthen our conclusions about the MOT model's lack of reliability. However, increasing the complexity of the sexual structure of the dynamical model would have little influence on the comparison between the benchmark and cMOT models, because the cMOT would be adjusted accordingly.

The interventions simulated from 2012 onwards, and the resource-allocation examples, were illustrative. However, they provide a useful warning for the potential implication of MOT-guided policies, and to highlight the need for policies to consider long-term impact. More detailed modelling and cost-effectiveness analysis would be required to make intervention-specific recommendations for policy-decisions.

### Summary and Recommendations

The generic MOT model remains the current template for HIV epidemic appraisals [9]. Our findings suggest that the reliability of the MOT metric could be improved by either using locally calibrated dynamical models for generating the MOT metric, or using parameters from calibrated dynamical models in the static MOT model (akin to the ‘unbiased’ inputs for the cMOT used here). We could also use alternate short-term metrics, such as the fraction of new HIV infections transmitted from a given subgroup (i.e. the “MOT transmitted”), or the annual PAF generated from dynamical models. However, none of these solutions address the fundamental issue that short-term estimates of HIV acquisition or transmission inherently underestimate the long-term contribution of epidemic drivers to overall HIV transmission under most conditions (except during the early epidemic phase). As the main problem with the MOT model rests with the inadequacy of the MOT metric, rather than model specification, the validity of the MOT metric in identifying epidemic drivers cannot be improved by calibrating the MOT model (using ‘unbiased’ inputs), increasing the complexity of the MOT model structure, or using a dynamical model to generate the metric.

We conclude that the MOT metric, in and of itself, is not a valid stand-alone tool and should not be used for selecting HIV prevention targets because it consistently underestimates the contribution of epidemic drivers to overall HIV transmission in the medium- to long-term. Translation of the MOT metric into policy could fail to reach epidemic drivers, and lead to less effective HIV prevention. Additional tools to characterize HIV epidemics that are based on a new paradigm of taking a long-term view (such as the long-term PAF) and that try to identify epidemic drivers are required, and their objective validation is necessary prior to wide-scale use.

## Supporting Information

Figure S1 **Schematic of HIV progression, combination antiretroviral (cART) treatment, and discontinuation.** Dashed arrows represent the excess mortality due to HIV. Symbols correspond to Equations 1-7 in Text S1 and Table S1. cART (combination antiretroviral treatment).
(TIF)

Figure S2 **Synthetic concentrated epidemic using data from Belgaum, south India.** Depicts subgroup HIV prevalence in the concentrated epidemic as predicted by the 4-stage and 1-stage dynamical models and the observed HIV prevalence (Belgaum, India) from available data sources that were used for the 4-stage DM calibration to generate the synthetic concentrated epidemic. The 1-stage dynamical model (DM) assumes uniform HIV infectivity throughout an individual's HIV infection, while the 4-stage DM incorporates differential HIV infectivity by stage of infection. The vertical capped lines represent 95% confidence intervals from the observed HIV prevalence. Note that different scales on the y-axis are used in A and B. ANC (antenatal clinic surveillance); GPS (general population survey); FSW (female sex worker). *ANC data was adjusted using 2007 GPS and ANC data comparison.
(TIF)

Figure S3 **Synthetic generalized epidemic using data from Lesotho.** Depicts subgroup HIV prevalence in the generalized epidemic as predicted by the 4-stage and 1-stage dynamical models (DM) and the observed HIV prevalence (Lesotho) from available data sources that were used for the 4-stage DM model calibration to generate the generalized epidemic. The 1-stage DM assumes uniform HIV infectivity throughout an individual's HIV infection, while the 4-stage DM incorporates differential HIV infectivity by stage of infection. The vertical capped lines represent 95% confidence intervals from the observed HIV prevalence. Note that different scales on the y-axis are used in Panels A and B. ANC (antenatal clinic surveillance); DHS (demographic health survey).
(TIF)

Figure S4 **Synthetic mixed epidemic using data from Kisumu, Kenya.** Depicts subgroup HIV prevalence in the mixed epidemic as predicted by the 4-stage and 1-stage dynamical models (DM) and the observed HIV prevalence (Kisumu, Kenya) from available data sources that were used for the 4-stage DM calibration to generate the synthetic mixed epidemic. The 1-stage DM assumes uniform HIV infectivity throughout an individual's HIV infection, while the 4-stage DM incorporates differential HIV infectivity by stage of infection. The vertical capped lines represent 95% confidence intervals from the observed HIV prevalence. Note that different scales on the y-axis are used in Panels A and B. ANC (antenatal clinic surveillance).
(TIF)

Figure S5 **Epidemic curves for the synthetic concentrated, generalized, and mixed epidemics.** The epidemic that would manifest in absence of commercial sex (dashed line) or the absence of casual sex (solid line with circles) is depicted alongside the full epidemic curve (solid line). In the generalized epidemic (B), commercial sex has little direct or indirect impact on HIV prevalence. In the mixed epidemic (C), commercial and casual sex both contribute to sustained transmission. Note that different scales on the y-axis are used in each panel.
(TIF)

Figure S6 **The MOT metric over time in the concentrated epidemic, by model type.** The Modes of Transmission (MOT) metric was measured every 5 years: (A) benchmark MOT (A); (B) MOT metric from the complex MOT model; (C) MOT metric from the generic MOT model (C). The MOT metric reflects the fraction of new HIV infections acquired by different risk groups (colored bars) estimated for 2012 using data from the synthetic epidemics. Early in the epidemic, most new infections occurred among clients and FSWs. As the epidemic progressed, and in the presence of increasing condom-use within high-risk partnerships, most new HIV infections occurred in the low-activity group.
(TIF)

Figure S7 **The MOT metric over time in the generalized epidemic, by model type.** The Modes of Transmission (MOT) metric was measured every 5 years: (A) benchmark MOT (A); (B) MOT metric from the complex MOT model; (C) MOT metric from the generic MOT model (C). The MOT metric reflects the fraction of new HIV infections acquired by different risk groups (colored bars) estimated for 2012 using data from the synthetic epidemics. Early in the epidemic, most new infections occurred among individuals in the multiple partner (MP) group. As the epidemic progressed, and in the presence of increasing condom-use within high-risk partnerships, most new HIV infections occurred in near equal proportions between the low-activity group and the MP groups (benchmark MOT [A] and the MOT metric from the complex MOT [B]).
(TIF)

Figure S8 **The MOT metric over time in the mixed epidemic, by model type.** The Modes of Transmission (MOT) metric was measured every 5 years: (A) benchmark MOT (A); (B) MOT metric from the complex MOT model; (C) MOT metric from the generic MOT model (C). The MOT metric reflects the fraction of new HIV infections acquired by different risk groups (colored bars) estimated for 2012 using data from the synthetic epidemics. Early in the epidemic, most new infections occurred among individuals engaged in multiple partnerships (MP). As the epidemic progressed, and in the presence of increasing condom-use within high-risk partnerships, most new HIV infections occurred in the low-activity group.
(TIF)

Figure S9 **Sensitivity of the MOT metric to biased input parameters (generalized epidemic).** The range in the predicted fraction of new HIV infections acquired by the low-activity group (A), clients (B), and individuals engaged in multiple partnerships (MP, C) are depicted for the five most influential parameters from the complex Modes of Transmission model (cMOT) using biased inputs. Also shown are the benchmark MOT metric and the unbiased cMOT metric. In the parameter range examined here, the biased cMOT model identified the epidemic driver (red regions) when (i) the number of sex-acts/year in a main partnership was underestimated; or (ii) the number of sex-acts/year in a casual partnership was overestimated. Pop. (population); Prev. (prevalence).
(TIF)

Figure S10 **Sensitivity of the MOT metric to biased input parameters (mixed epidemic).** The range in the predicted fraction of new HIV infections acquired by the low-activity group (A), clients (B), female sex workers (FSWs, C), and individuals with multiple partnerships (D) are depicted for the five most influential parameters from the complex Modes of Transmission model (cMOT) using biased inputs. Also shown are the benchmark MOT metric and the unbiased cMOT metric. Across the parameter range examined here, the low-activity group incurred the largest burden of new infections, and the unbiased MOT metric did not identify the epidemic driver (no red regions). Pop. (population); Prev. (prevalence); MP (multiple partnership group).
(TIF)

Figure S11 **MOT metrics by subgroups and their contribution to overall HIV transmission (generalized epidemic).** The predicted fraction of new infections acquired by individuals with multiple partnerships (A, MP) and the low-activity group (B), as obtained from the complex Modes of Transmission model (cMOT acquired) and the generic Modes of Transmission model (gMOT acquired), and the benchmark MOT (acquired), are shown in grey. The fraction of HIV infections transmitted from FSWs and the low-activity group is shown in green (cMOT transmitted). The cumulative population attributable fraction (PAF_t_) over different time horizons measured from the year of the MOT (2012) for the epidemic driver (FSWs) and low-activity groups are shown in black.
(TIF)

Figure S12 **MOT metrics by subgroups and their contribution to overall HIV transmission (mixed epidemic).** The predicted fraction of new infections acquired by female sex workers (A, FSWs) and the low-activity group (B), as obtained from the complex Modes of Transmission model (cMOT acquired) and the generic Modes of Transmission model (gMOT acquired), and the benchmark MOT (acquired), are shown in grey. The fraction of HIV infections transmitted from FSWs and the low-activity group is shown in green (cMOT transmitted). The cumulative population attributable fraction (PAF_t_) over different time horizons measured from the year of the MOT (2012) for the epidemic driver (FSWs) and low-activity groups are shown in black.
(TIF)

Table S1 **State variables and parameters for the dynamical and MOT models.**
(DOCX)

Text S1 **Dynamical and MOT model details.**
(DOCX)
