# Supplementary figures and images for: Validation of the Modes of Transmission Model as a Tool to Prioritize HIV Prevention Targets: A Comparative Modelling Analysis
Source: PLoS One. 2014 Jul 9;9(7):e101690. doi: 10.1371/journal.pone.0101690 (PMC4090151; doi:10.1371/journal.pone.0101690)

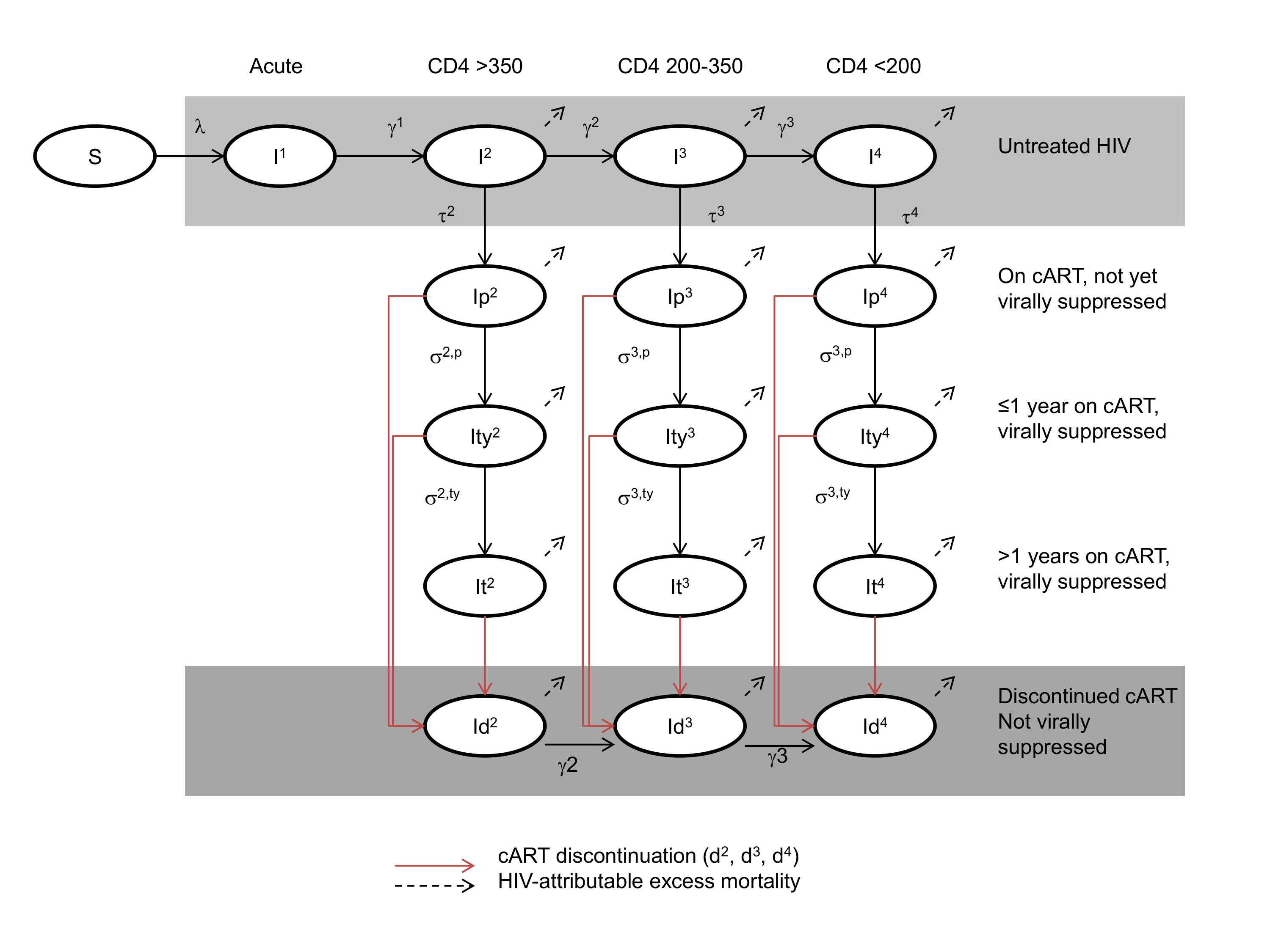

Supplement: Figure S1 — Schematic of HIV progression, combination antiretroviral (cART) treatment, and discontinuation. Dashed arrows represent the excess mortality due to HIV. Symbols correspond to Equations 1-7 in Text S1 and Table S1. cART (combination antiretroviral treatment). (TIF) [file pone.0101690.s001.tif]

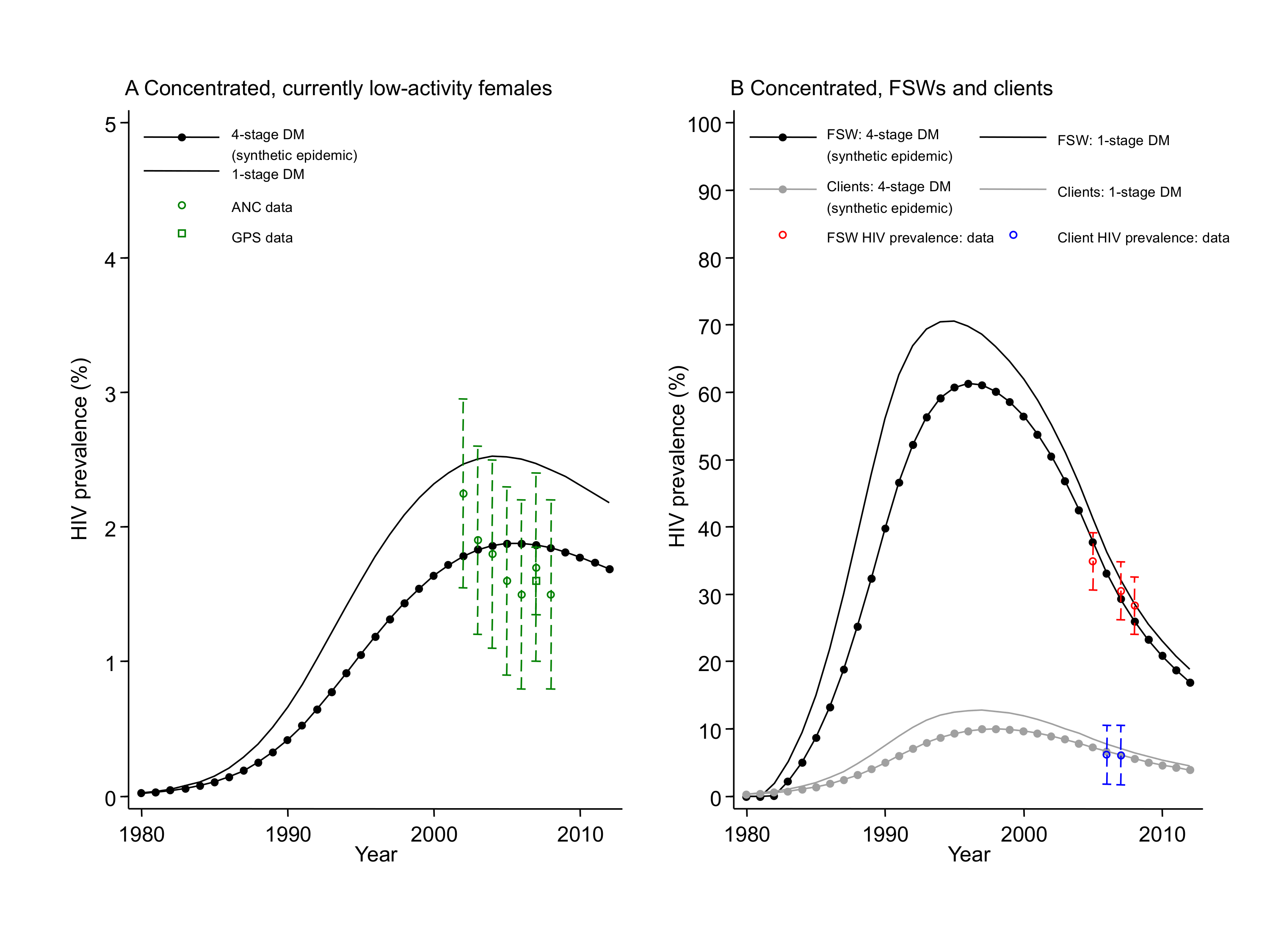

Supplement: Figure S2 — Synthetic concentrated epidemic using data from Belgaum, south India. Depicts subgroup HIV prevalence in the concentrated epidemic as predicted by the 4-stage and 1-stage dynamical models and the observed HIV prevalence (Belgaum, India) from available data sources that were used for the 4-stage DM calibration to generate the synthetic concentrated epidemic. The 1-stage dynamical model (DM) assumes uniform HIV infectivity throughout an individual's HIV infection, while the 4-stage DM incorporates differential HIV infectivity by stage of infection. The vertical capped lines represent 95% confidence intervals from the observed HIV prevalence. Note that different scales on the y-axis are used in A and B. ANC (antenatal clinic surveillance); GPS (general population survey); FSW (female sex worker). *ANC data was adjusted using 2007 GPS and ANC data comparison. (TIF) [file pone.0101690.s002.tif]

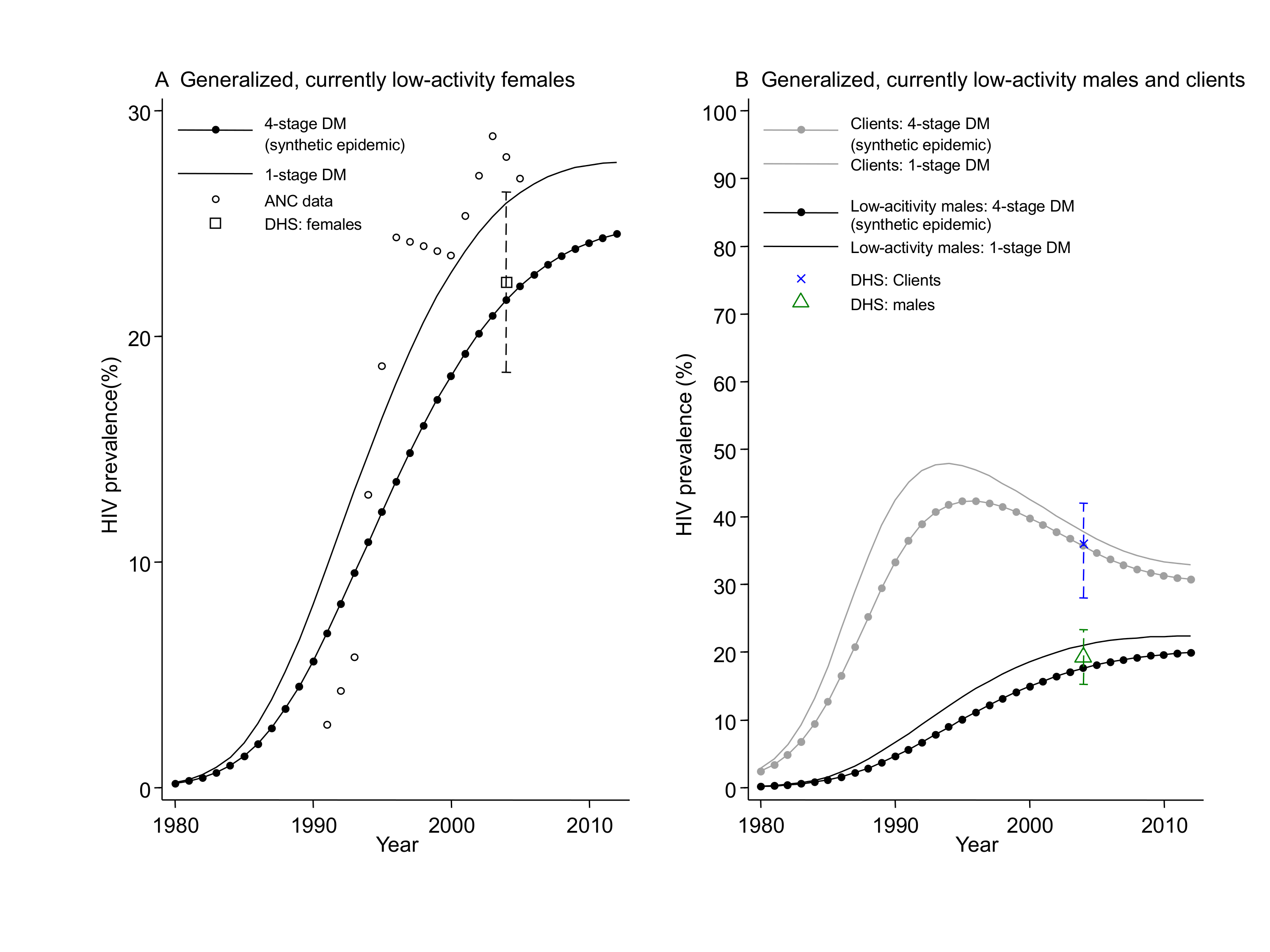

Supplement: Figure S3 — Synthetic generalized epidemic using data from Lesotho. Depicts subgroup HIV prevalence in the generalized epidemic as predicted by the 4-stage and 1-stage dynamical models (DM) and the observed HIV prevalence (Lesotho) from available data sources that were used for the 4-stage DM model calibration to generate the generalized epidemic. The 1-stage DM assumes uniform HIV infectivity throughout an individual's HIV infection, while the 4-stage DM incorporates differential HIV infectivity by stage of infection. The vertical capped lines represent 95% confidence intervals from the observed HIV prevalence. Note that different scales on the y-axis are used in Panels A and B. ANC (antenatal clinic surveillance); DHS (demographic health survey). (TIF) [file pone.0101690.s003.tif]

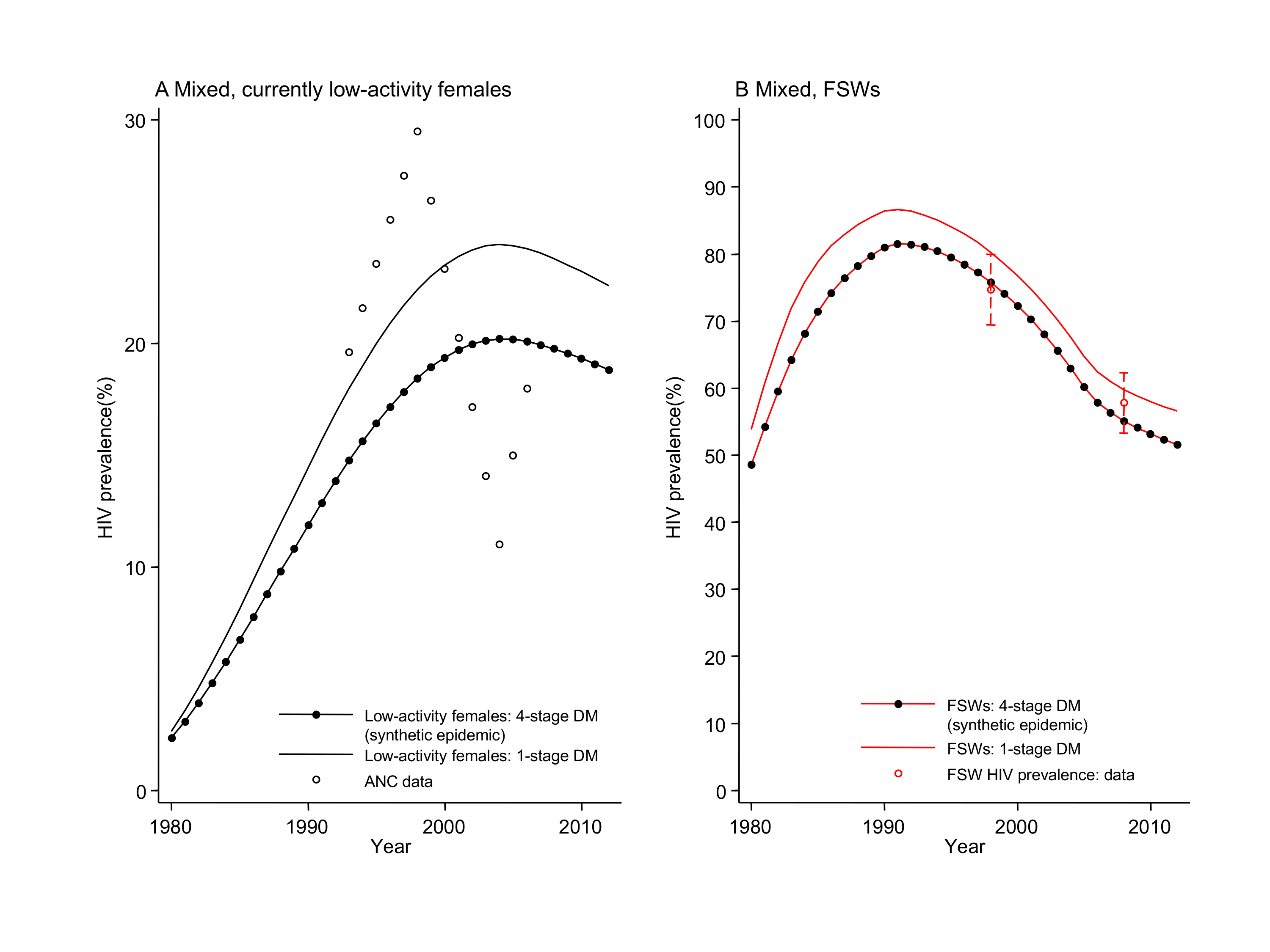

Supplement: Figure S4 — Synthetic mixed epidemic using data from Kisumu, Kenya. Depicts subgroup HIV prevalence in the mixed epidemic as predicted by the 4-stage and 1-stage dynamical models (DM) and the observed HIV prevalence (Kisumu, Kenya) from available data sources that were used for the 4-stage DM calibration to generate the synthetic mixed epidemic. The 1-stage DM assumes uniform HIV infectivity throughout an individual's HIV infection, while the 4-stage DM incorporates differential HIV infectivity by stage of infection. The vertical capped lines represent 95% confidence intervals from the observed HIV prevalence. Note that different scales on the y-axis are used in Panels A and B. ANC (antenatal clinic surveillance). (TIF) [file pone.0101690.s004.tif]

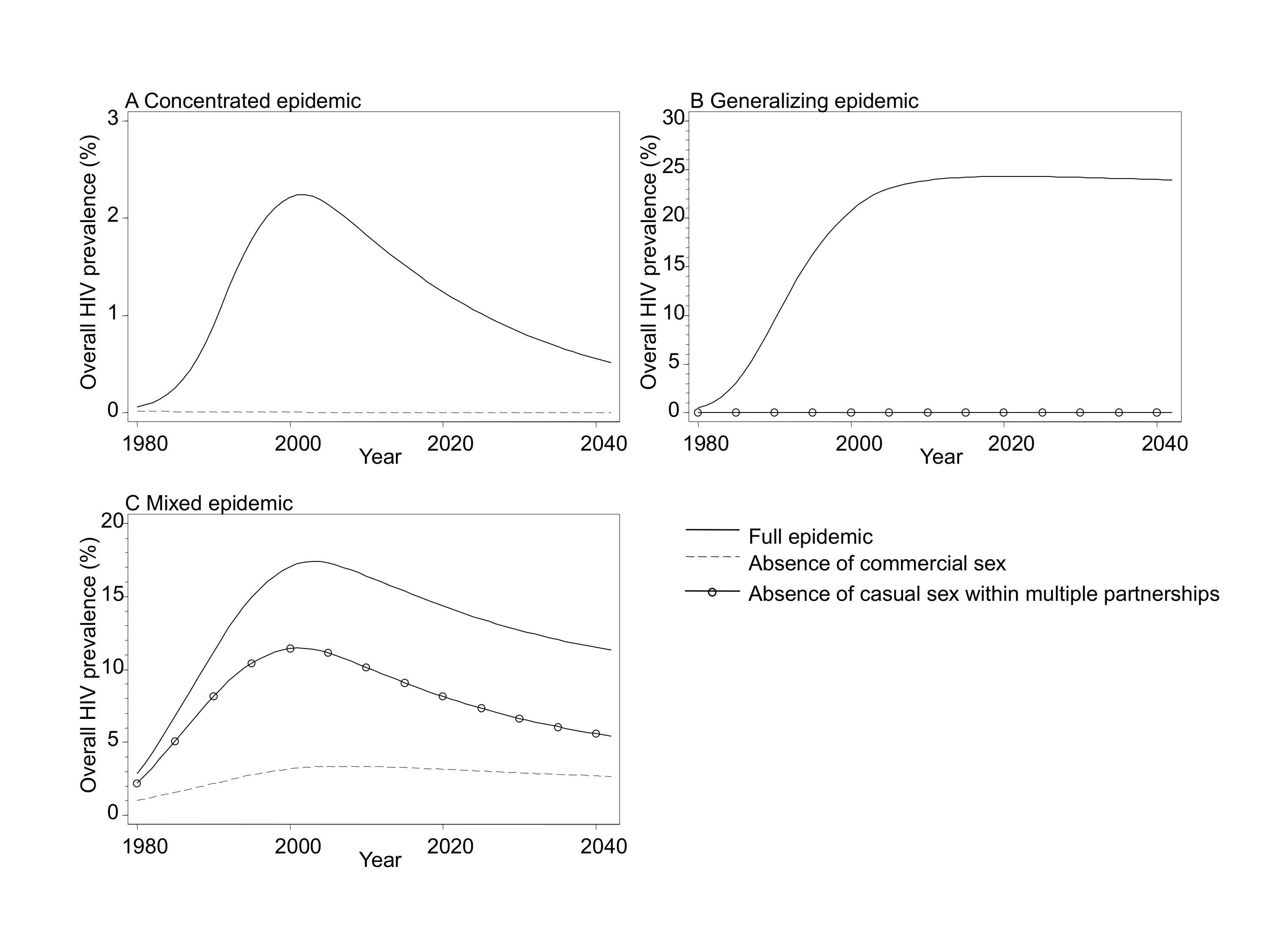

Supplement: Figure S5 — Epidemic curves for the synthetic concentrated, generalized, and mixed epidemics. The epidemic that would manifest in absence of commercial sex (dashed line) or the absence of casual sex (solid line with circles) is depicted alongside the full epidemic curve (solid line). In the generalized epidemic (B), commercial sex has little direct or indirect impact on HIV prevalence. In the mixed epidemic (C), commercial and casual sex both contribute to sustained transmission. Note that different scales on the y-axis are used in each panel. (TIF) [file pone.0101690.s005.tif]

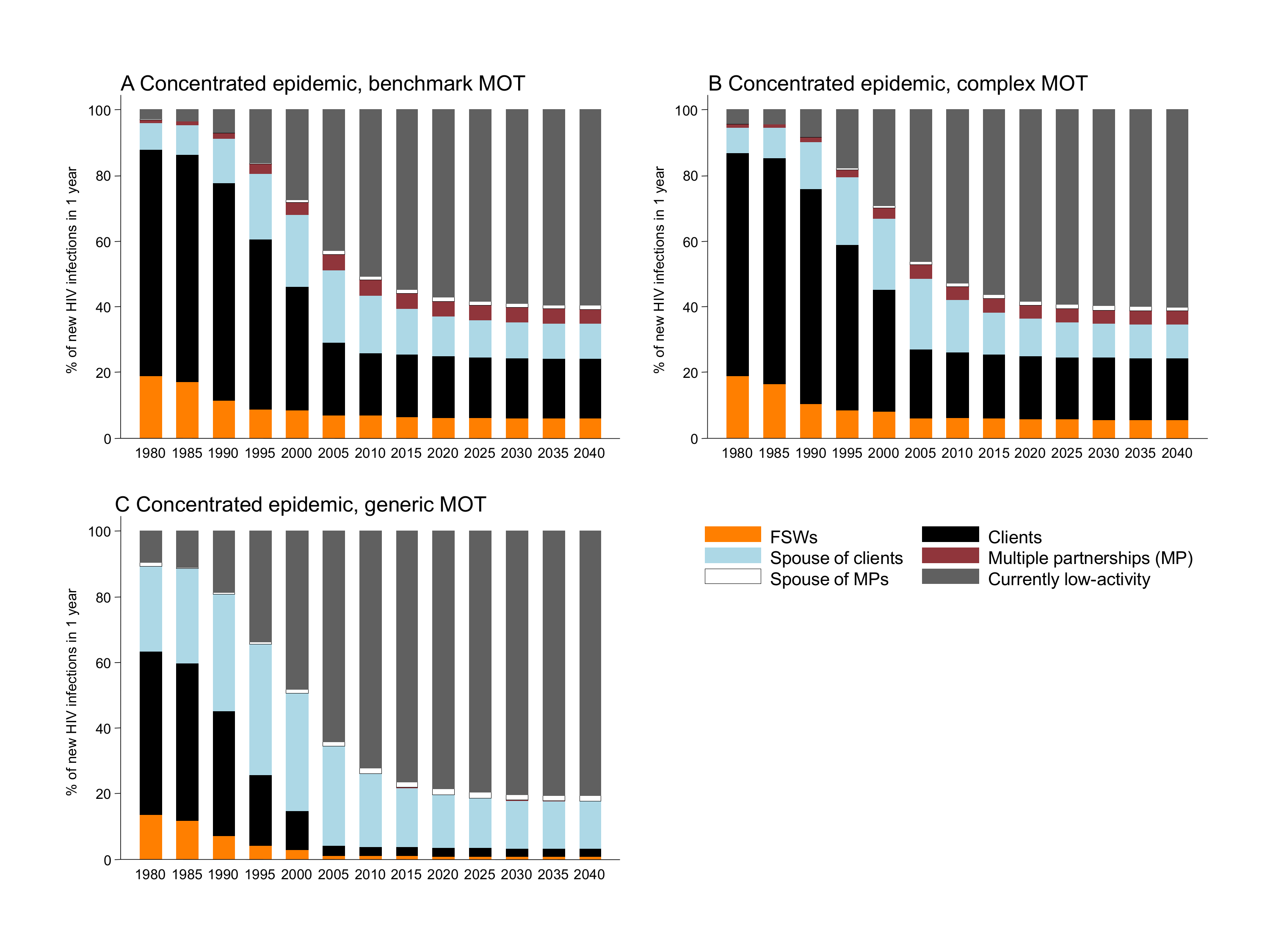

Supplement: Figure S6 — The MOT metric over time in the concentrated epidemic, by model type. The Modes of Transmission (MOT) metric was measured every 5 years: (A) benchmark MOT (A); (B) MOT metric from the complex MOT model; (C) MOT metric from the generic MOT model (C). The MOT metric reflects the fraction of new HIV infections acquired by different risk groups (colored bars) estimated for 2012 using data from the synthetic epidemics. Early in the epidemic, most new infections occurred among clients and FSWs. As the epidemic progressed, and in the presence of increasing condom-use within high-risk partnerships, most new HIV infections occurred in the low-activity group. (TIF) [file pone.0101690.s006.tif]

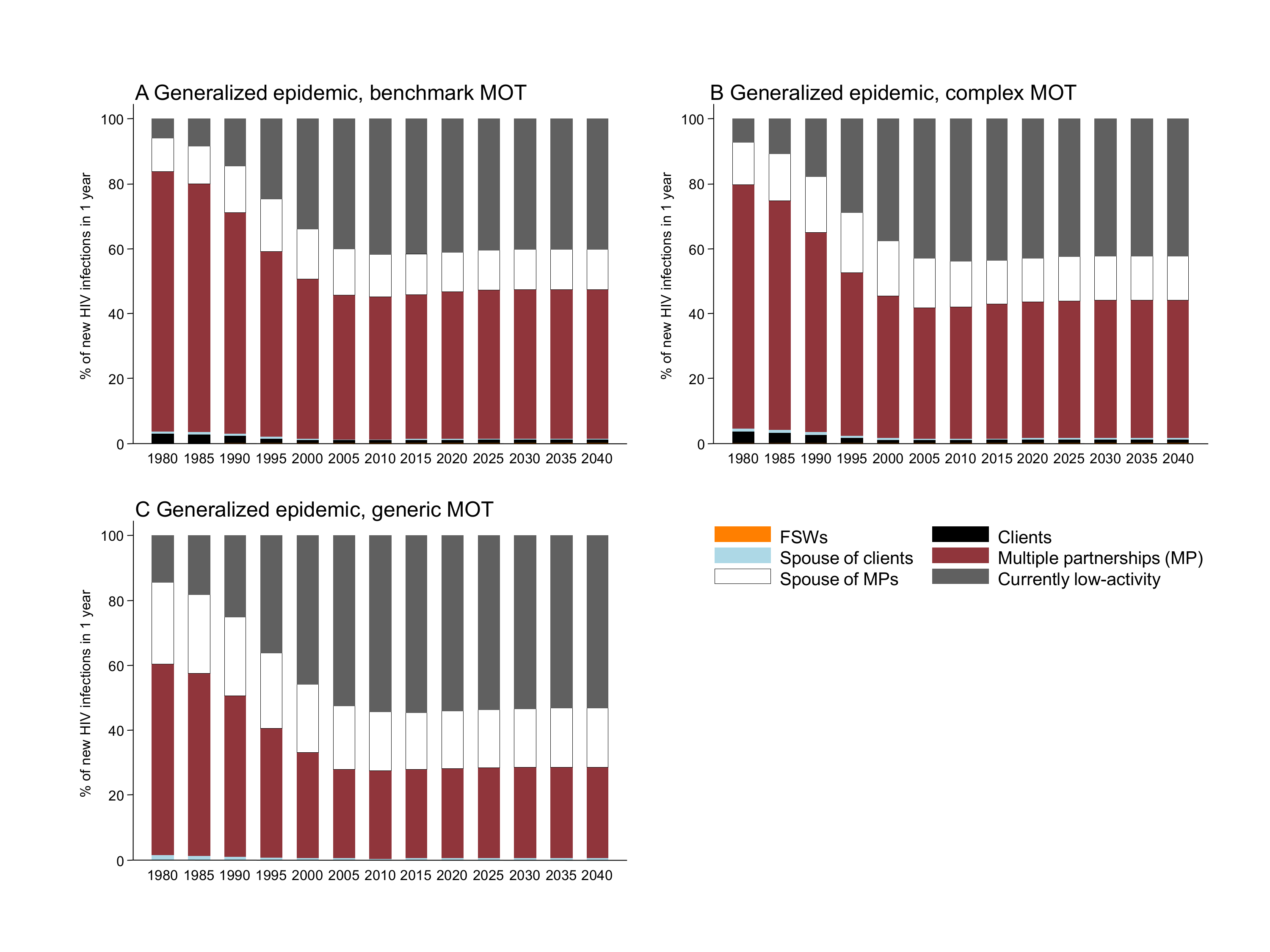

Supplement: Figure S7 — The MOT metric over time in the generalized epidemic, by model type. The Modes of Transmission (MOT) metric was measured every 5 years: (A) benchmark MOT (A); (B) MOT metric from the complex MOT model; (C) MOT metric from the generic MOT model (C). The MOT metric reflects the fraction of new HIV infections acquired by different risk groups (colored bars) estimated for 2012 using data from the synthetic epidemics. Early in the epidemic, most new infections occurred among individuals in the multiple partner (MP) group. As the epidemic progressed, and in the presence of increasing condom-use within high-risk partnerships, most new HIV infections occurred in near equal proportions between the low-activity group and the MP groups (benchmark MOT [A] and the MOT metric from the complex MOT [B]). (TIF) [file pone.0101690.s007.tif]

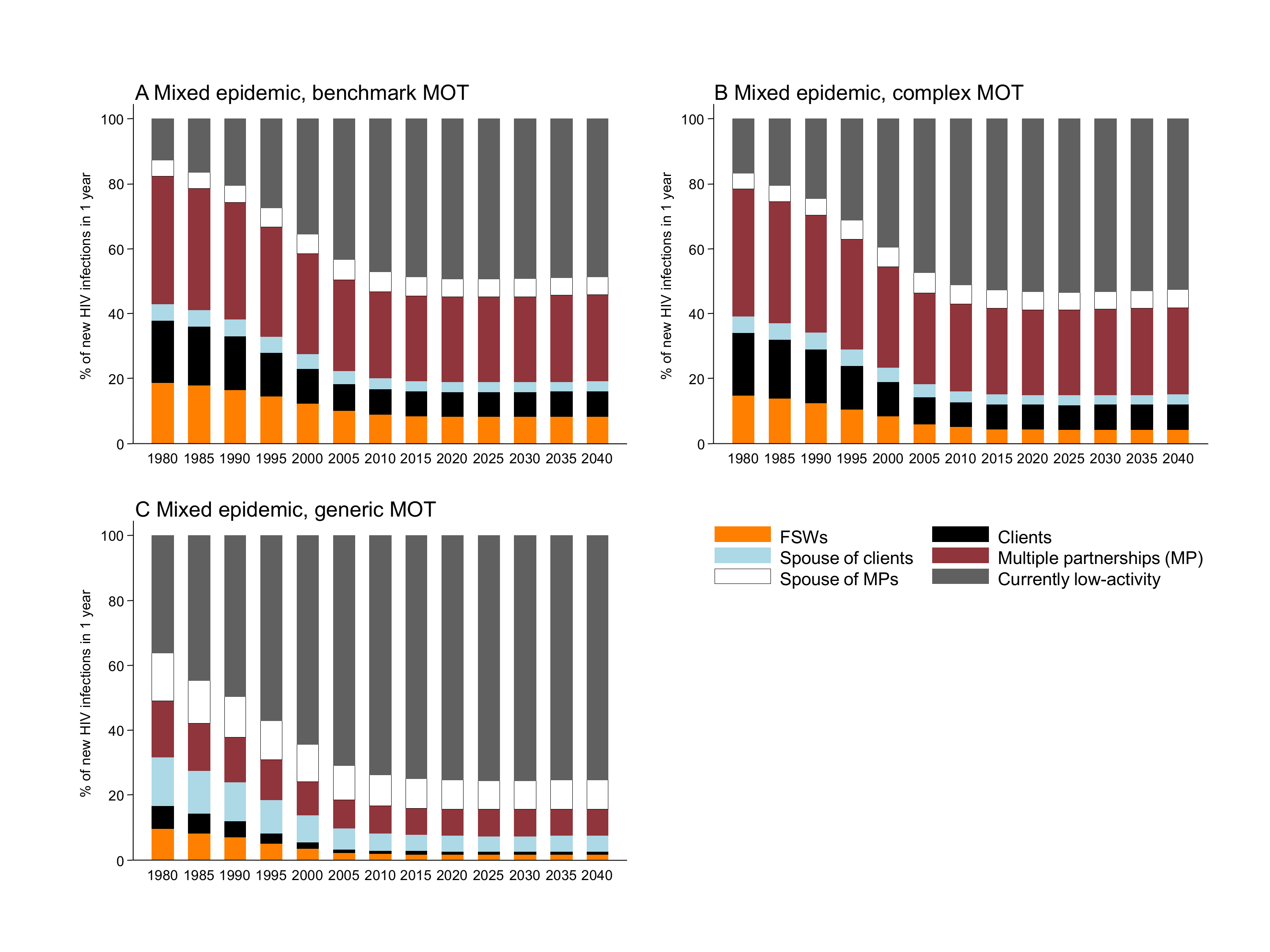

Supplement: Figure S8 — The MOT metric over time in the mixed epidemic, by model type. The Modes of Transmission (MOT) metric was measured every 5 years: (A) benchmark MOT (A); (B) MOT metric from the complex MOT model; (C) MOT metric from the generic MOT model (C). The MOT metric reflects the fraction of new HIV infections acquired by different risk groups (colored bars) estimated for 2012 using data from the synthetic epidemics. Early in the epidemic, most new infections occurred among individuals engaged in multiple partnerships (MP). As the epidemic progressed, and in the presence of increasing condom-use within high-risk partnerships, most new HIV infections occurred in the low-activity group. (TIF) [file pone.0101690.s008.tif]

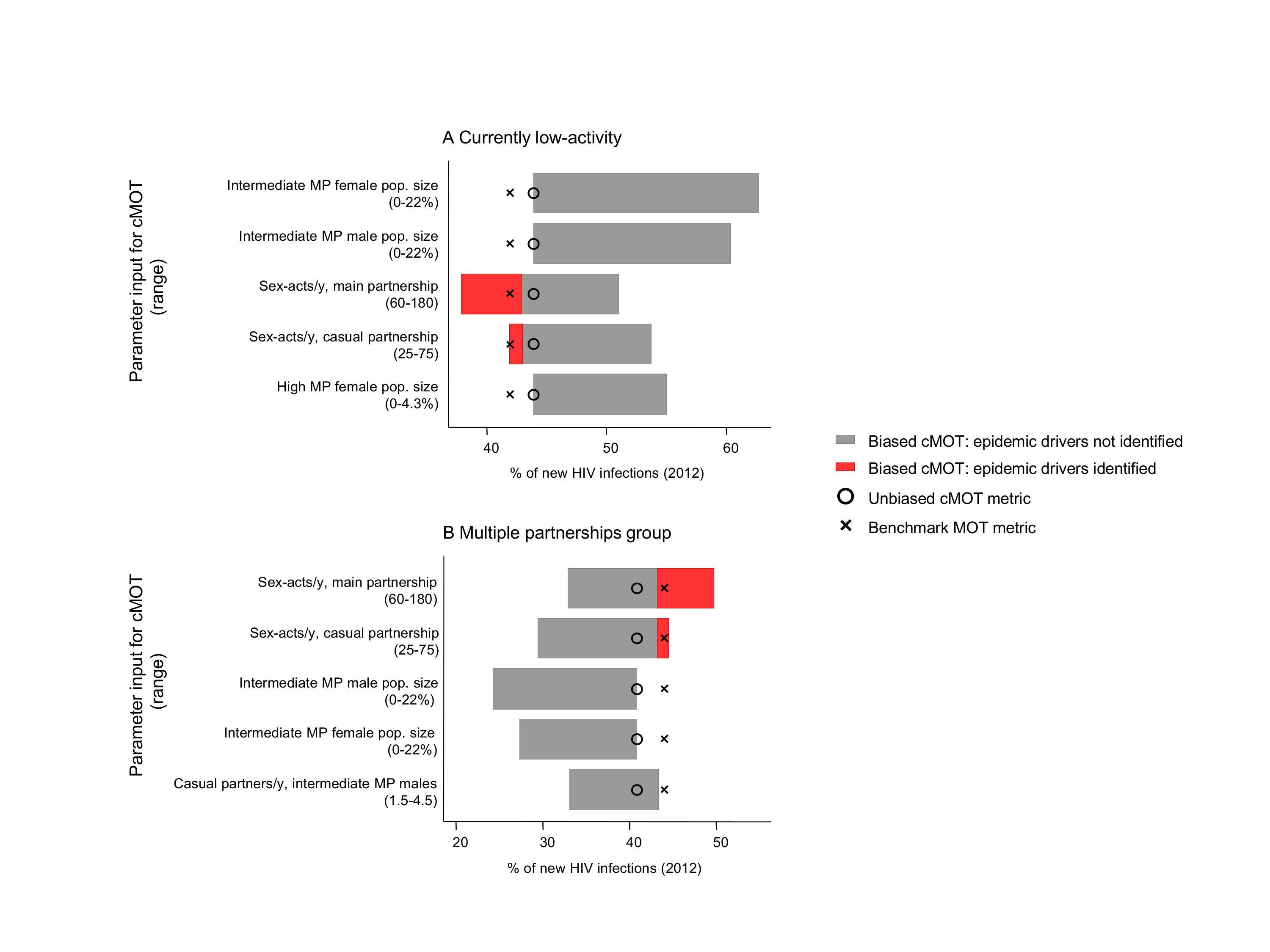

Supplement: Figure S9 — Sensitivity of the MOT metric to biased input parameters (generalized epidemic). The range in the predicted fraction of new HIV infections acquired by the low-activity group (A), clients (B), and individuals engaged in multiple partnerships (MP, C) are depicted for the five most influential parameters from the complex Modes of Transmission model (cMOT) using biased inputs. Also shown are the benchmark MOT metric and the unbiased cMOT metric. In the parameter range examined here, the biased cMOT model identified the epidemic driver (red regions) when (i) the number of sex-acts/year in a main partnership was underestimated; or (ii) the number of sex-acts/year in a casual partnership was overestimated. Pop. (population); Prev. (prevalence). (TIF) [file pone.0101690.s009.tif]

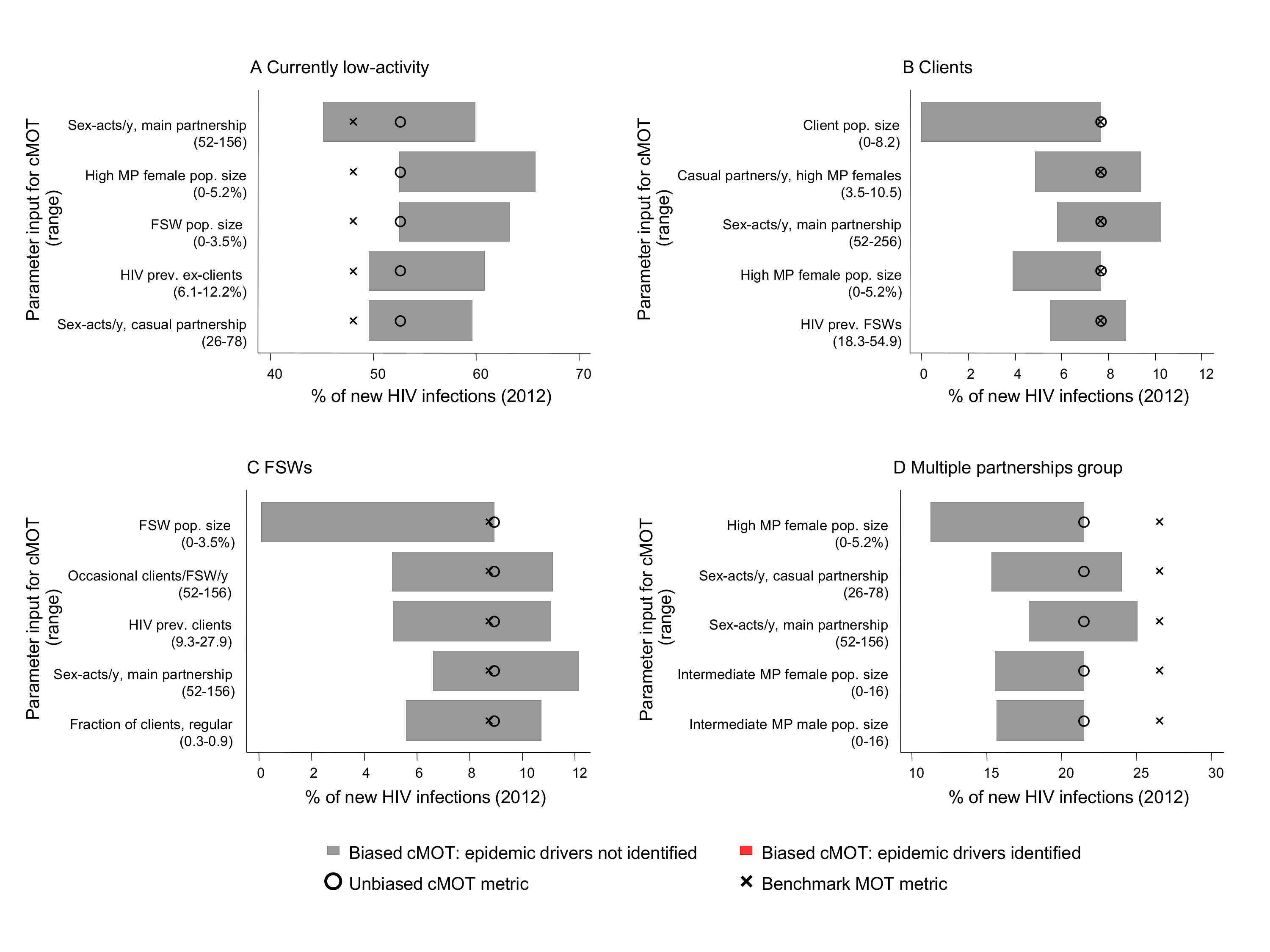

Supplement: Figure S10 — Sensitivity of the MOT metric to biased input parameters (mixed epidemic). The range in the predicted fraction of new HIV infections acquired by the low-activity group (A), clients (B), female sex workers (FSWs, C), and individuals with multiple partnerships (D) are depicted for the five most influential parameters from the complex Modes of Transmission model (cMOT) using biased inputs. Also shown are the benchmark MOT metric and the unbiased cMOT metric. Across the parameter range examined here, the low-activity group incurred the largest burden of new infections, and the unbiased MOT metric did not identify the epidemic driver (no red regions). Pop. (population); Prev. (prevalence); MP (multiple partnership group). (TIF) [file pone.0101690.s010.tif]

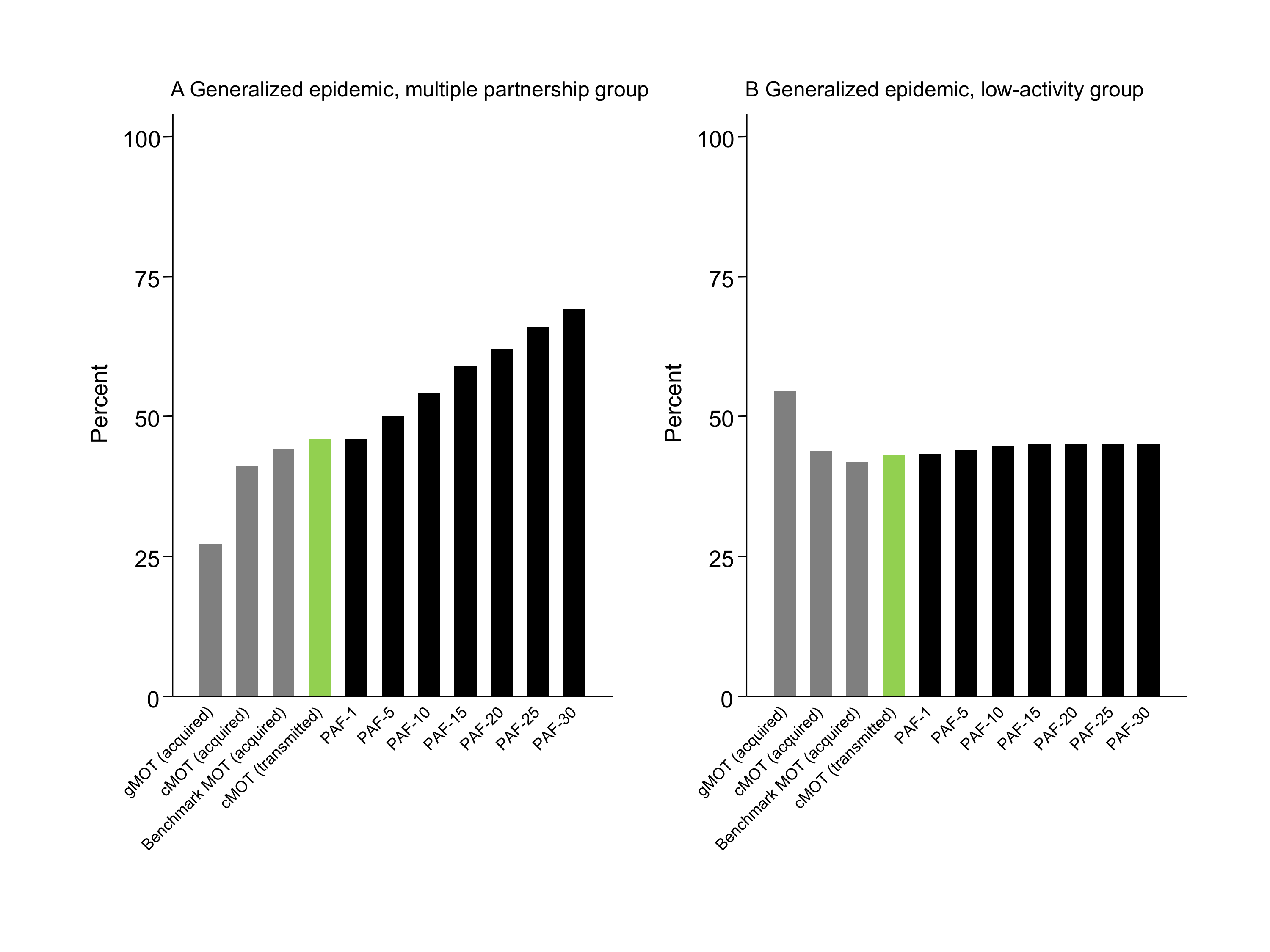

Supplement: Figure S11 — MOT metrics by subgroups and their contribution to overall HIV transmission (generalized epidemic). The predicted fraction of new infections acquired by individuals with multiple partnerships (A, MP) and the low-activity group (B), as obtained from the complex Modes of Transmission model (cMOT acquired) and the generic Modes of Transmission model (gMOT acquired), and the benchmark MOT (acquired), are shown in grey. The fraction of HIV infections transmitted from FSWs and the low-activity group is shown in green (cMOT transmitted). The cumulative population attributable fraction (PAFt) over different time horizons measured from the year of the MOT (2012) for the epidemic driver (FSWs) and low-activity groups are shown in black. (TIF) [file pone.0101690.s011.tif]

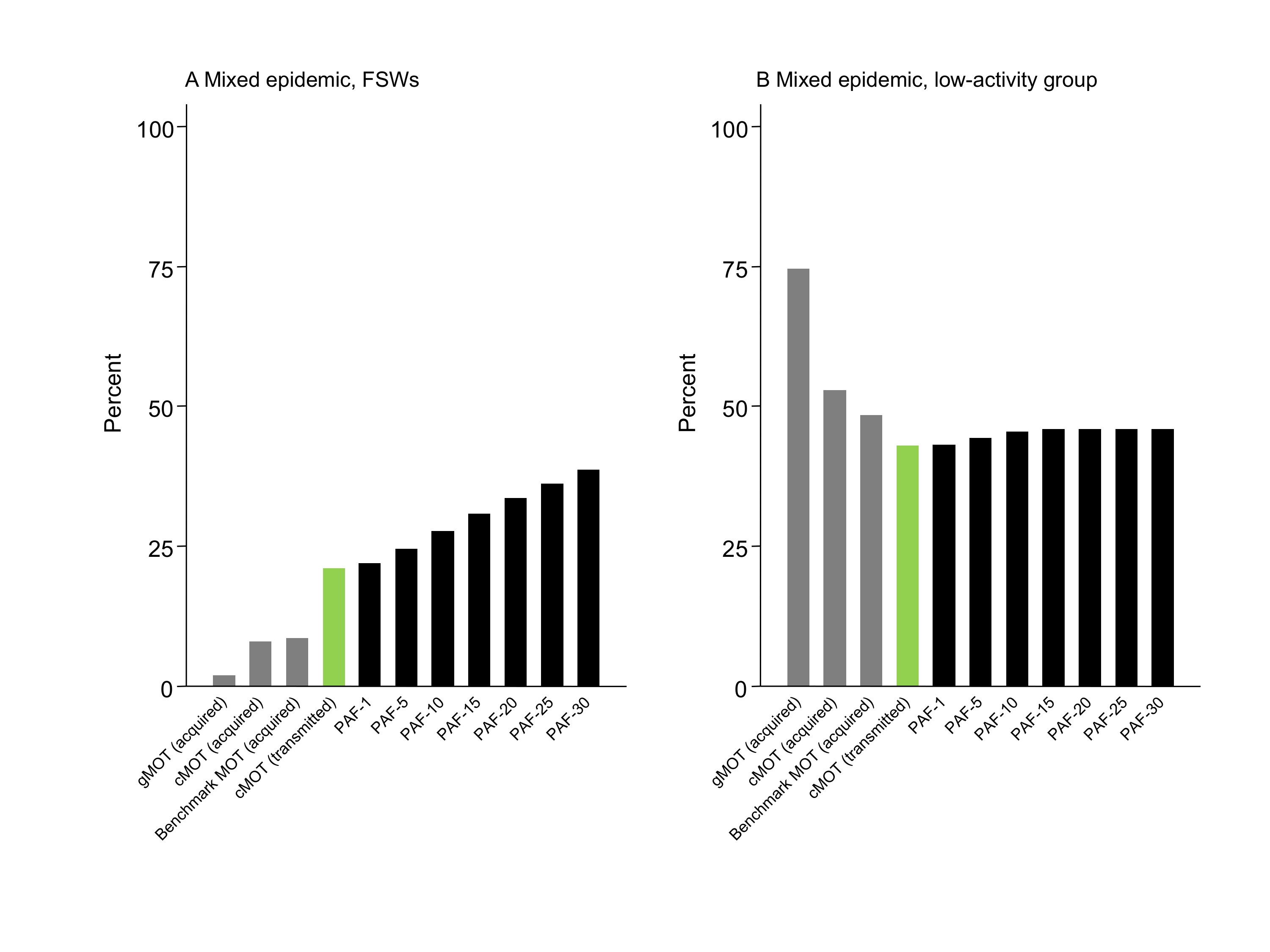

Supplement: Figure S12 — MOT metrics by subgroups and their contribution to overall HIV transmission (mixed epidemic). The predicted fraction of new infections acquired by female sex workers (A, FSWs) and the low-activity group (B), as obtained from the complex Modes of Transmission model (cMOT acquired) and the generic Modes of Transmission model (gMOT acquired), and the benchmark MOT (acquired), are shown in grey. The fraction of HIV infections transmitted from FSWs and the low-activity group is shown in green (cMOT transmitted). The cumulative population attributable fraction (PAFt) over different time horizons measured from the year of the MOT (2012) for the epidemic driver (FSWs) and low-activity groups are shown in black. (TIF) [file pone.0101690.s012.tif]
