# Supplementary material for: Validation of the Modes of Transmission Model as a Tool to Prioritize HIV Prevention Targets: A Comparative Modelling Analysis
Source: PLoS One. 2014 Jul 9;9(7):e101690. doi: 10.1371/journal.pone.0101690 (PMC4090151; doi:10.1371/journal.pone.0101690)
Supplement: Table S1 — State variables and parameters for the dynamical and MOT models. (DOCX) [file pone.0101690.s013.docx]

**Table S1. State variables and parameters for the dynamical and MOT models.**

| **Symbol** | **Parameter/variable descriptor** | **Value** |
| --- | --- | --- |
| **Subscripts and superscripts** |  |  |
| *i* | Subscript for gender class | 1=male  2=female |
| *j* | Subscript for sexual activity class | 1=commercial  2=high-frequency multiple partnerships  3=intermediate frequency multiple partnerships  4=always low-activity group  5=previously engaged in commercial sex  6=previously high-frequency multiple partnerships  7=previously intermediate-frequency multiple partnerships  *l* = {4,5,6,7} |
| *z* | Stage of HIV | 1= acute HIV  2 = asymptomatic (CD4 >350)  3 = pre-AIDS (CD4 200-350)  4 = CD4 <200 |
| *π* | Type of partnership | 1=regular commercial  2=occasional commercial  3=casual  4=main |
| **State variables** |  |  |
| S_ij_ | Susceptible to HIV (sexually active) | State variable |
| I^z^ | HIV-infected, and treatment naive  I^1^ (acute stage)  I^2^ (CD4>350 cells/μL)  I^3^ (CD4200-350 cells/μL)  I^4^ (CD4≤200 cells/μL) | State variable  j∈{1,2,3,*l*} |
| Ip^z^ | HIV-infected, initiated on cART, and not yet virally suppressed  Ip^1^ (acute stage)  Ip^2^ (CD4>350 cells/μL)  Ip^3^ (CD4200-350 cells/μL)  Ip^4^ (CD4≤200 cells/μL) | State variable  j∈{1,2,3,*l*} |
| Ity^z^ | HIV-infected, first year on cART after viral suppression  Ity^1^ (acute stage)  Ity^2^ (CD4>350 cells/μL)  Ity^3^ (CD4200-350 cells/μL)  Ity^4^ (CD4≤200 cells/μL) | State variable  j∈{1,2,3,*l*} |
| It^z^ | HIV-infected, >1 year on cART after viral suppression  It^1^ (acute stage)  It^2^ (CD4>350 cells/μL)  It^3^ (CD4200-350 cells/μL)  It^4^ (CD4≤200 cells/μL) | State variable  j∈{1,2,3,*l*} |
| Id^z^ | HIV-infected, no longer on effective cART due to self-discontinuation of cART, loss to follow-up, or virological treatment failure  Id^1^ (acute stage)  Id^2^ (CD4>350 cells/μL)  Id^3^ (CD4200-350 cells/μL)  Id^4^ (CD4≤200 cells/μL) | State variable  j∈{1,2,3,*l*} |
| **Transition rates and related parameters** |  |  |
| $N_{i}^{tot}$ | Total starting population (15-49 years of age) in each gender  (the initial population in 1975 was extrapolated from the 2011 population (Belgaum and Lesotho) or 2006 population (Kisumu), by using the linear growth rate,  *pr*). | 2011 population  Belgaum[1] (projected population from 2001 census):  Females (1,180,389)  Males (1,243,092)  Lesotho[2]:  Females(654,700)  Males (634,198)  Kisumu county[3]:  Females(298,681)  Males (286,717) |
| *pr* | Annual crude birth rate[2]  Rate assumed to remain unchanged | Belgaum: 2.2% (India data)  Lesotho: 2.8%  Kisumu: 3.7% (Kenya data) |
| $\omega_{ij}$ | Number of individuals entering into each activity class *j* | Eqn (1) |
| $\zeta$ _21_ | Rate of entering commercial sex among female sex workers (per-capita) | 1/duration of sex work  (see table 1 main text) |
| $\zeta$ _11_ | Rate of entering into commercial sex among clients (per-capita) | 1/duration of paid sex among clients  (see table 1 main text) |
| $\zeta$ _i2_, $\zeta$ _i3_ | Rate of entering into the multiple partnerships class (per-capita) | 1/duration of higher-risk sex among MP class  (see table 1 main text) |
| $Q_{ij}$ | Fraction of individuals who enter each higher-activity class, calibrated to the following values in year 2005:  $\frac{N_{ij}}{N_{i}^{tot}}=F_{ij(time=2005)}$ for j∈{1,3},i∈{1,2}  $\frac{N_{ij}}{N_{i}^{tot}}=F_{ij(time=2005)}$ for j∈{2},i=2  $\frac{N_{i2}}{N_{i}^{tot}}=F_{i2(time=2005)}-[{{prob}_{ij}^{\pi=3}F}_{11(time=2005)}]$ for j∈{2},i=1 | See Table 1 main text for $F_{ij (time=2005)}$ |
| $\zeta$ _i4_ | Rate of entering into the low activity class (per-capita) | 1/duration of sexual activity  See table 1 main text |
| $\varepsilon_{ij}$ | Rate of moving from higher-activity to lower-activity group (per-capita) | j∈{1-3}; $\zeta$ _ij_  j∈{4-7}; 0 |
| $\mu_{ij}$ | Rate of ceasing to be sexually active (per-capita) | j∈{1,3}; 0  j=4; $\zeta$ _i4_  j∈{5,7}; $\left( \frac{1}{\mu_{i4}}-\frac{1}{\varepsilon_{ij-4}} \right)^{-1}$years^-1^ |
| $\gamma^{z}=\left( \frac{1}{{dur}^{z}} \right)-\phi^{z}$ | Rate of progression from HIV stage z to stage z+1  Nb: z=4; 0 |  |
| ${dur}^{z}$ | Average duration of time (years) spent in each HIV stage, z, before progression to stage z+1 or dying due to HIV-attributable mortality. | z=1; 0.21[4]  z=2; 4.8 [5,6]  z=3; 3.7 [5,6]  z=4; 2.0[5] |
| $\phi^{z}$ | HIV-attributable mortality rate (per-capita) in the following 3 groups: untreated, discontinued treatment, by stage of HIV prior to treatment initiation  z=1; 0  z=2; 5% [[12](#_ENREF_12)]  z=3; 10.4% [5]  z=4; 50% [5] | Assumption of no excess mortality during acute stage HIV (z=1). Excess HIV-attributable mortality before CD4 decline reflects AIDS defining illnesses (such as TB and bactermia [5,7]). |
| $\phi^{z,p}, \phi^{z,ty}$ | HIV-attributable mortality rate (per-capita) during first year on treatment.  z=2; 1.6%[7]  z=3; 5%[8]  z=4; 10%[8,9] | Assumption that even before viral load suppression is achieved following the start of cART, a reduction in HIV-attributable mortality is expected due to diagnosis and treatment of concomitant disease and prevention of opportunistic infections (eg. pneumocystis *jirovicii* pneumonia prophylaxis for stage z=4). However, excess mortality during the 1^st^ year of treatment is higher than subsequent years on treatment. cART is not started in the acute stage of HIV. |
| $\phi^{z,tr}$ | HIV-attributable mortality rate (per-capita, per year) after the first year on treatment and while virally suppressed[[12](#_ENREF_12)]  z=2; 0.8%  z=3; 2.5%  z=4; 5% | Excess mortality following the 1^st^ year of treatment is assumed to occur at a constant rate. Excess mortality after the 1^st^ year on treatment falls to half the excess mortality rate during the 1^st^ year on treatment. |
| $\sigma^{z,p}$ | 1/time from cART initiation to viral load suppression  6 months (irrespective of pre-treatment CD4 [z]) |  |
| $\sigma^{z,ty}$ | 1/time from viral load suppression to end of first year on treatment  6 months |  |
| $\tau_{ij}^{z}$ | cART initiation rate (a combination of HIV testing, linkage to care, and initiation of cART either due to CD4 eligibility or stage 3-4 HIV) $\tau_{ij}^{1}=0$  $\tau_{ij}^{2}=0$  $\tau_{ij}^{3}=0.1$ (concentrated), 0.3 (generalizing), 0.2 (mixed)  $\tau_{ij}^{4}=0$.3 (concentrated), 0.3 (generalizing), 0.2 (mixed) | Calibrated to overall 2011 ART coverage and CD4 distribution at ART initiation. [10-12] |
| $d_{ij}^{z,p}$, $d_{,ij}^{z,ty}, d_{ij}^{z,tr}$ | cART discontinuation rate (treatment failure, or self-discontinuation, or persistent non-adherence), per-capita per year  for all z, treatment state (p, ty, tr): 5%[9] | Assumes a constant discontinuation rate and no 2^nd^ line treatment, no viral load monitoring, ∴ no re-initiation of cART |
| **Other parameters** |  |  |
| *α_π_* | Number of sex acts per year within each partnership type | See table 1 man text |
| $C_{ij\pi}$ | Yearly partner change rate for each type of partnership  $C_{ij4}$  remainder | 1  See table 1 main text |
| ρ_ii’111_ | Probability of a regular commercial sex partner among clients and FSWs | See table 1 main text  i'≠i |
| ρ_ii’112_ | Probability of an occasional commercial sex partner among clients and FSWs | 1  i'≠i |
| ρ_ii’j1π_ | Probability of a commercial sex partner among non-FSWs and non-clients.  π∈{1,2} and j≠1 | 0  i'≠i |
| ${prob}_{ij}^{\pi=3}$ | Probability an individual has a casual partner | i=1 and j=1; 0  π∈{1,2} and j∈{4-7};0  All others, see table 1 main text |
| C_ijπ_ | Partner exchange rate among individuals in sex i, activity class j, for partnership type π | See table 1 main text |
| $\beta_{12}^{2,u}$ | Probability of transmission per sex act from female to male, when the female partner is not virally suppressed (during asymptomatic, CD4>350, stage). | (1-mc*eff_mc)*0.00043[13,14] |
| *mc* | Proportion of males that are circumcised | Belgaum:0%  Lesotho:10%[15]  Kisumu: 25%[12] |
| *eff_mc* | Efficacy of male circumcision in reducing HIV susceptibility among HIV-negative males | 0.6 |
| $\beta_{21}^{2,u}$ | Probability of transmission per sex act from male to female, when the male partner is not virally suppressed (during asymptomatic, CD4>350, stage) | 0.00065[13,14] |
| $\beta_{ii^{'}}^{z,u}=rr\_z\beta_{ii^{'}}^{2,u}$ | Relative increase in per-act transmission probability  Acute (z=1)  Asymptomatic (z=2)  Pre-AIDS, 200-350 cells/mm^3^ (z=3)  AIDS or <200 cells/mm^3^ (z=4) | rr_1=5[13,16]  rr_2=1  rr_3=1.9[13,16]  rr_4=5[13,16]  For the 1-stage dynamical model and each static MOT model, the weighted average of the per-act transmission probability was used |
| *rr^s^* | Relative increase in HIV susceptibility due to a concomitant HSV-2 and/or genital ulcer disease. Per sex act (while shedding). 20% of HSV-2 seropositive individuals are assumed to be shedding at any time | 2.0[17-21] |
| *rr^i^* | Relative increase in HIV infectivity due to a concomitant HSV-2 and/or genital ulcer disease. Per sex act (while shedding). 20% of HSV-2 seropositive individuals are assumed to be shedding at any time | 2.0[14,21,22] |
| *eff_condom* | Efficacy of condoms in reducing HIV transmission per sex act. | 85%[14] |
| $\kappa_{\pi}$ | Condom coverage by partnership type π | See table 1 and 3 main text |
| $\tau_{ij}^{z}$ | Rate of initiating cART  z=1; 0 for all i,j (no treatment for acute HIV)  calibrated to overall cART coverage by 2011 in each representative region[12,23,24] | See Eqn (45) |
| *eff_art* | Efficacy of combination anti-retroviral treatment in reducing HIV transmission per sex act, assuming viral load suppression | 0.96[25] |

MOT (modes of transmission).

**References**

1. Government of India Ministry of Home Affairs, Registrar General & Census Commissioner India (2011) Census india. Available: http://www.censusindia.gov.in/ Accessed 1 December 2013

2. The World Bank Databank (2011). Available: http://data.worldbank.org/indicator/SP.POP.GROW

3. Kenya National Bureau Of Statistics (2013). Kenya population statistics. Available: http://www.knbs.or.ke/ Accessed 1 September 2013

4. Cohen MS, Shaw GM, Mcmichael AJ, Haynes BF (2011) Medical progress: Acute HIV-1 infection. N Engl J Med 364: 1943-1954

5. Anglaret X, Minga A, Gabillard D, Ouassa T, Messou E, et al. (2012) AIDS and non-AIDS morbidity and mortality across the spectrum of CD4 cell counts in HIV-infected adults before starting antiretroviral therapy in Cote d'Ivoire. Clin Infect Dis 54: 714-723. doi: 10.1093/cid/cir898

6. Lodi S, Phillips A, Touloumi G, Geskus R, Meyer L, et al. (2011) Time from human immunodeficiency virus seroconversion to reaching CD4+ cell count thresholds < 200, < 350, and < 500 cells/mm^3^: Assessment of need following changes in treatment guidelines. Clin Infect Dis 53: 817-825. doi: 10.1093/cid/cir494

7. Kitahata MM, Gange SJ, Abraham AG, Merriman B, Saag MS, et al. (2009) Effect of early versus deferred antiretroviral therapy for hiv on survival. N Engl J Med 360: 1815-1826. doi: 10.1056/NEJMoa0807252

8. Etard JF, Ndiaye I, Thierry-Mieg M, Gueye NFN, Gueye PM, et al. (2006) Mortality and causes of death in adults receiving highly active antiretroviral therapy in Senegal: A 7-year cohort study. AIDS 20: 1181-1189. doi: 10.1097/01.aids.0000226959.87471.01

9. Mahy M, Lewden C, Brinkhof MWG, Dabis F, Tassie J-M, et al. (2010) Derivation of parameters used in spectrum for eligibility for antiretroviral therapy and survival on antiretroviral therapy. Sex Transm Infect 86(Suppl 2): ii28-ii34

10. UNAIDS (2013) Report on the global AIDS epidemic. Geneva. Available: http://www.unaids.org/en/resources/campaigns/20121120_globalreport2012/ Accessed 1 December 2013

11. India Health Action Trust (2010) HIV/AIDS situation and response in Karnataka: Epidemiological appraisal using data triangulation. Bangalore. Available: http://www.ihat.in/Documents.html Accessed 1 December 2013

12. National Aids Control Council (2013) Kenya ungass country progress report 2012. Geneva. Available: http://www.unaids.org/en/dataanalysis/knowyourresponse/countryprogressreports/2012countries/ Accessed 1 July 2013

13. Boily MC, Baggaley RF, Wang L, Masse B, White RG, et al. (2009) Heterosexual risk of HIV-1 infection per sexual act: Systematic review and meta-analysis of observational studies. Lancet Infect Dis 9: 118-129

14. Hughes JP, Baeten JM, Lingappa JR, Magaret AS, Wald A, et al. (2012) Determinants of per-coital-act HIV-1 infectivity among African HIV-1-serodiscordant couples. J Infect Dis 205: 358-365. doi: 10.1093/infdis/jir747

15. Khobotlo M, Tshehlo R, Nkonyana J, Ramoseme M, Khobotle M, et al. (2009) Lesotho HIV prevention response and modes of transmission analysis. Available: http://www.unaidsrstesa.org/thematic-areas/hiv-prevention/know-your-epidemic-modes-transmission Accessed 1 Mar 2013

16. Donnell D, Baeten JM, Kiarie J, Thomas KK, Stevens W, et al. (2010) Heterosexual HIV-1 transmission after initiation of antiretroviral therapy: A prospective cohort analysis. Lancet 375: 2092-2098. doi: 10.1016/s0140-6736(10)60705-2

17. Freeman EE, Weiss HA, Glynn JR, Cross PL, Whitworth JA, et al. (2006) Herpes simplex virus 2 infection increases HIV acquisition in men and women: Systematic review and meta-analysis of longitudinal studies. AIDS 20: 73-83. doi: 10.1097/01.aids.0000198081.09337.a7

18. Wald A, Link K (2002) Risk of human immunodeficiency virus infection in herpes simplex virus type 2-seropositive persons: A meta-analysis. J Infect Dis 185: 45-52. doi: 10.1086/338231

19. Barnabas RV, Wasserheit JN, Huang YD, Janes H, Morrow R, et al. (2011) Impact of herpes simplex virus type 2 on HIV-1 acquisition and progression in an HIV vaccine trial (the STEP study). J Acquir Immune Defic Syndr 57: 238-244. doi: 10.1097/QAI.0b013e31821acb5

20. Chen L, Jha P, Stirling B, Sgaier SK, Daid T, et al. (2007) Sexual risk factors for HIV infection in early and advanced HIV epidemics in Sub-Saharan Africa: systematic overview of 68 epidemiological studies. PLoS One 2. doi: 10.1371/journal.pone.0001001

21. Serwadda D, Gray RH, Sewankambo NK, Wabwire-Mangen F, Chen MZ, et al. (2003) Human immunodeficiency virus acquisition associated with genital ulcer disease and herpes simplex virus type 2 infection: A nested case-control study in Rakai, Uganda. J Infect Dis 188: 1492-1497. doi: 10.1086/379333

22. Celum C, Wald A, Lingappa JR, Magaret AS, Wang RS, et al. (2010) Acyclovir and transmission of HIV-1 from persons infected with HJV-1 and HSV-2. N Engl J Med 362: 427-439. doi: 10.1056/NEJMoa0904849

23. Karnataka State AIDS Society (2008-2012) Annual action plan 2008-2009; 2009-2010; 2010-2011. Bangalore. Available: http://karhfw.gov.in/ksaps/index.html

24. Ministry of Health And Social Welfare (2012) Global AIDS response country progress report 2011: Lesotho. Available: http://www.unaids.org/en/dataanalysis/knowyourresponse/countryprogressreports/2012countries/ Accessed 1 July 2013

25. Cohen MS, Chen YQ, Mccauley M, Gamble T, Hosseinipour MC, et al. (2011) Prevention of HIV-1 infection with early antiretroviral therapy. N Engl J Med 365: 493-505. doi: 10.1056/NEJMoa1105243
