# Supplementary material for: Validation of the Modes of Transmission Model as a Tool to Prioritize HIV Prevention Targets: A Comparative Modelling Analysis
Source: PLoS One. 2014 Jul 9;9(7):e101690. doi: 10.1371/journal.pone.0101690 (PMC4090151; doi:10.1371/journal.pone.0101690)
Supplement: Text S1 — Dynamical and MOT model details. (DOCX) [file pone.0101690.s014.docx]

**Supplementary Text S1 and Table S1: Dynamical and MOT model details**

**1. Dynamical model**

1.1 Model structure & assumptions

We developed a deterministic, compartmental mathematical model of heterosexual HIV transmission, and solved the system of ordinary differential equations using the Runge-Kutta 4 numerical integration method in Berkeley Madonna. A schematic representation of the partnerships is shown in Figure 1 of the main text and a schematic representation HIV progression, treatment, and discontinuation of treatment is shown in the Supplementary Figure S1. The list of state variables and parameters are given below (Supplementary Table S1).

The model subdivides the population according to gender (males *i*=1, females *i*=2), and discrete higher-activity (male clients [*j*=1], female sex workers [FSW, *j*=1], high frequency of multiple partnerships [MP,*j*=2], intermediate frequency of MP [*j*=3]) and low-activity classes (individuals who had never engaged in higher-activity sex in their past [*j*=4], individuals who had previously engaged in sex work [*j*=5], individuals who engaged in high-frequency multiple partnerships [*j*=6], and individuals who engaged in intermediate-frequency multiple partnerships [*j*=7] in their past). Upon start of sexual activity, males and females enter one of four activity classes (*j*∈{1,2,3,4}). After a specified duration of time spent in a higher-activity activity classes (*j*∈{1-3}), individuals retire into their respective low-activity class (*j*∈{5,6,7}). To stabilize the relative size of subgroups before introducing HIV, the model is run with a 100-year burn in period. HIV infection is seeded with 50 infectious individuals in each class in 1975.

Individuals are susceptible (S) upon start of sexual activity, and become infected with HIV (acute stage, I^1^) with a force of infection (HIV incidence per susceptible) dependent on partnership type, frequency of partner change, and HIV prevalence of the partners. Individuals then progress (at rates γ) through a total of 4 (*z*) stages of untreated HIV (I^2^, I^3^, I^4^) reflecting the following: CD4 >350 cells/μL, CD4 between 200-350 cells/μL, and CD4 <200 cells/μL respectively. The natural history of HIV in the absence and presence of treatment is depicted in Supplementary Figure S1.

If patients are started on combination anti-retroviral treatment (cART) with rate $\tau_{ij}^{z}$, they enter a short period on treatment but not yet virally suppressed (Ip^1^,Ip^2^,Ip^3^,Ip^4^), followed by viral suppression during the first year of treatment (Ity^1^,Ity^2^,Ity^3^,Ity^4^), and viral suppression during the remainder of their time on treatment while still sexually active (It^1^,It^2^,It^3^,It^4^). After starting treatment, individuals experience a discontinuation rate (*d*, reflecting self-discontinuation, treatment failure, or loss to follow-up), and enter a separate state where they are no longer virally suppressed (Id^1^,Id^2^,Id^3^,Id^4^). Transition from Ip^z^ to Ity^z^ is given by σ^z,p^ while transition from Ity^z^ to It^z^ is given by σ^z,ty^. The HIV-attributable or excess mortality rate varies across the different treatment states. cART is assumed to reduce HIV-attributable mortality (φ), more so after the 1st year of treatment. cART is assumed to increase linearly from the first year of roll-out (setting-specific) to achieve the coverage (% of adults with HIV alive and on ART) by 2011. Following discontinuation or treatment failure, we assume that 2^nd^ line treatment is not available and patients are not re-initiated on first line treatment.

Co-infection with a sexually transmitted infection (to mimic herpes simplex virus-2 [HSV-2]) is included at a stable prevalence. HSV-2 is assumed to increase HIV infectiousness and susceptibility, per sex-act. Baseline male circumcision is included, as is baseline condom-use (assumed to have increased linearly to the last estimate of condom-use available [2005 for Lesotho and Kisumu, 2008 for Belgaum], where levels remain constant for the baseline scenarios).

Sexual activity does not change by HIV stage. Individuals leave this system (or population under study) upon cessation of all sexual activity or HIV-attributable mortality (μ). The relative size of subgroups changes as a result of HIV-attributable mortality, and partnerships are adjusted accordingly [1]. Partnership formation and dissolution is instantaneous, and therefore this model does not take into account duration of partnerships.

1.2 Model equations

The state variables are given by:

$\frac{dS_{ij}}{dt}=\omega_{ij}- \left( \mu_{ij}+ \varepsilon_{ij} \right)S_{ij}- \lambda_{ij}S_{ij}$ (1)

$\frac{{dI}_{ij}^{1}}{dt}= \delta_{ij}^{1}-\left( \mu_{ij}+\varepsilon_{ij}+ \phi^{1}+ \gamma^{1}+ \tau_{ij}^{1} \right)I_{ij}^{1} + \lambda_{ij}S_{ij}$ (2)

$\frac{{dI}_{ij}^{z}}{dt}= \delta_{ij}^{z}-\left( \mu_{ij}+\varepsilon_{ij}+ \phi^{z}+ \gamma^{z}+ \tau_{ij}^{z} \right)I_{ij}^{z}+ \gamma^{z-1}I_{ij}^{z-1} ;z\in\{2,3,4\}$ (3)

$\frac{{dIp}_{ij}^{z}}{dt}= {\delta p}_{ij}^{z}-\left( \mu_{ij}+\varepsilon_{ij}+ \phi^{z,p}+ \sigma^{z,p}+d_{ij}^{z,p} \right){Ip}_{ij}^{z} + \tau_{ij}^{z}I_{ij}^{z} ;z\in\{1,2,3,4\}$ (4)

$\frac{{dIty}_{ij}^{z}}{dt}= {\delta ty}_{ij}^{z}-\left( \mu_{ij}+\varepsilon_{ij}+ \phi^{z,ty}+ \sigma^{z,ty}+d_{ij}^{z,ty} \right){Ity}_{ij}^{z} + \sigma^{z,p}{Ip}_{ij}^{z} ;z\in\{1,2,3,4\}$ (5)

$\frac{{dIt}_{ij}^{z}}{dt}= {\delta t}_{ij}^{z}-\left( \mu_{ij}+\varepsilon_{ij}+ \phi^{z,tr}+ d_{ij}^{z,tr} \right){It}_{ij}^{z} + \sigma^{z,ty}{Ity}_{ij}^{z} ;z\in\{1,2,3,4\}$ (6)

$\frac{{dId}_{ij}^{z}}{dt}= {\delta d}_{ij}^{z}-\left( \mu_{ij}+\varepsilon_{ij}+ \phi^{z}+ \gamma^{z} \right){Id}_{ij}^{z}+ d_{ij}^{z,p}{Ip}_{ij}^{z}+ d_{ij}^{z,ty}{Ity}_{ij}^{z}+ d_{ij}^{z,tr}{It}_{ij}^{z}+ \gamma^{z-1}{Id}_{ij}^{z-1} ;z\in\{1,2,3,4\}$ (7)

The size of each subgroup is given by:

$N_{ij}=S_{ij}+ \sum_{x=1}^{4} I_{ij}^{x}+ \sum_{x=1}^{4} {Ip}_{ij}^{x}+ \sum_{x=1}^{4} {Ity}_{ij}^{x}+ \sum_{x=1}^{4} {It}_{ij}^{x}+ \sum_{x=1}^{4} {Id}_{ij}^{x}$ (8)

Where, for individuals entering into activity class *j* at a rate of $\omega_{ij}$:

$\omega_{ij}=\left\{ \begin{matrix} \zeta_{ij}P_{ij} \\ \varepsilon_{ij-4}S_{ij-4} \end{matrix} | \begin{matrix} j\in\left\{ 1,2,3,4 \right\} \\ j\in\left\{ 5,6,7 \right\} \end{matrix} \right\}$ (9)

And the initial population of each activity class ($P_{ij}$) is given by:

$P_{ij}=\left\{ \begin{matrix} {N_{i}^{tot}f}_{ij} \\ N_{i}^{tot}(1-\sum_{j'=1}^{j'=7} f_{ij'}) \\ 0 \end{matrix} | \begin{matrix} j\in\left\{ 1,2,3 \right\} \\ j=4, j\neq j^{'} \\ j\in\left\{ 5,6,7 \right\} \end{matrix} \right\}$ (10)

Where,

$f_{ij}=\left\{ \begin{matrix} Q_{ij} \\ \frac{N_{ij}}{N_{i}^{tot}} \end{matrix} | \begin{matrix} j\epsilon\left\{ 12,3 \right\} \\ j\epsilon\left\{ 4,5,6,7 \right\} \end{matrix} \right\}$ (11)

$N_{i}^{tot}=N_{i}^{1975}\left( 1+pr \right)$ after HIV is seeded into the population in 1975 (12)

Where *pr* = population growth rate (assumed to be the same for males and females)

The rate of movement from high to low-activity activity classes among individuals who have become infected, are given by:

$\delta_{ij}^{z}= \left\{ \begin{matrix} 0 \\ \varepsilon_{ij-4}I_{ij-4}^{z} \end{matrix} | \begin{matrix} j\in\left\{ 1,2,3,4 \right\} \\ j\in\left\{ 5,6,7 \right\} \end{matrix} \right\} ; z \in\{1,2,3,4\}$ (13)

${\delta p}_{ij}^{z}= \left\{ \begin{matrix} 0 \\ \varepsilon_{ij-4}{Ip}_{ij-4}^{z} \end{matrix} | \begin{matrix} j\in\left\{ 12,3,4 \right\} \\ j\in\left\{ 5,6,7 \right\} \end{matrix} \right\} ; z \in\{1,2,3,4\}$ ` (14)

${\delta ty}_{ij}^{z}= \left\{ \begin{matrix} 0 \\ \varepsilon_{ij-4}{Ity}_{ij-4}^{z} \end{matrix} | \begin{matrix} j\in\left\{ 12,3,4 \right\} \\ j\in\left\{ 5,6,7 \right\} \end{matrix} \right\} ; z \in\{1,2,3,4\}$ (15)

${\delta t}_{ij}^{z}= \left\{ \begin{matrix} 0 \\ \varepsilon_{ij-4}{It}_{ij-4}^{z} \end{matrix} | \begin{matrix} j\in\left\{ 12,3,4 \right\} \\ j\in\left\{ 5,6,7 \right\} \end{matrix} \right\} ; z \in\{1,2,3,4\}$ (16)

${\delta d}_{ij}^{z}= \left\{ \begin{matrix} 0 \\ \varepsilon_{ij-4}{Id}_{ij-4}^{z} \end{matrix} | \begin{matrix} j\in\left\{ 12,3,4 \right\} \\ j\in\left\{ 5,6,7 \right\} \end{matrix} \right\} ; z \in\{1,2,3,4\}$ (17)

In the sexual activity classes where j∈{4-7}form one pool of currently low-activity individuals, we group them together.

$S_{il}=\sum_{j=4}^{7} S_{ij}$

$N_{il}=\sum_{j=4}^{7} N_{ij}$

$I_{il}^{z}=\sum_{j=4}^{7} I_{ij}^{z}$

${Ip}_{il}^{z}=\sum_{j=4}^{7} {Ip}_{ij}^{z}$

${Ity}_{il}^{z}=\sum_{j=4}^{7} {Ity}_{ij}^{z}$

${It}_{il}^{z}=\sum_{j=4}^{7} {It}_{ij}^{z}$

${Id}_{il}^{z}=\sum_{j=4}^{7} {Id}_{ij}^{z}$ (18)

The sums of individuals who are infectious are divided into those who are virally suppressed, and those who are not virally suppressed.

Not virally suppressed:

${Iu}_{il}^{z}=I_{il}^{z}+ {Ip}_{il}^{z}+ {Id}_{il}^{z} ;j \in\left\{ 4,5,6,7 \right\}, z \in\{1,2,3,4\}$ (19)

$${Iu}_{ij}^{z}= I_{ij}^{z}+ {Ip}_{ij}^{z}+ {Id}_{ij}^{z} ; j \in\left\{ 1,2,3 \right\}, z \in\{1,2,3,4\}$$

Virally suppressed:

${Iv}_{il}^{z}={Ity}_{il}^{z}+ {It}_{il}^{z} ;j \in\left\{ 4,5,6,7 \right\}, z \in\{1,2,3,4\}$ (20)

${Iv}_{ij}^{z}= {Ity}_{ij}^{z}+ {It}_{ij}^{z} ;$ $j \in\left\{ 1,2,3 \right\}, z \in\{1,2,3,4\}$

The force of infection per susceptible individual is given by:

$\lambda_{ij}= \sum_{\pi=1}^{4} \sum_{j=1}^{l} \rho_{ii^{;}jj^{;}\pi}\sum_{z=1}^{4} \left\{ 1-\left[ \left( \frac{{Beta}_{ii^{'}jj^{'}\pi}^{z,u}{Iu}_{i^{'}j^{'}}^{x}+ {Beta}_{ii^{'}jj^{'}\pi}^{z,v}{Iv}_{i^{'}j^{'}}^{x}}{N_{i^{'}j^{'}}} \right)+\left( 1-\frac{\left( {Iu}_{i^{'}j^{'}}^{x}+{Iv}_{i^{'}j^{'}}^{x} \right)}{N_{i^{'}j^{'}}} \right) \right]^{C_{ij\pi}} \right\}$; *i≠ i’* and *j*∈ {1,2,3,*l*} (21)

Where:

${Beta}_{ii^{'}jj^{'}\pi}^{z,u}$ and ${Beta}_{ii^{'}jj^{'}\pi}^{z,v}$ represent the per-partnership transmission of HIV from infected individual in sex *i’* to opposite sex *i*, when the infected partner is in sexual activity class *j’* and HIV stage z, and the partnership is of the type π. *Beta^u^* reflects the per partnership transmission from partners who are not virally suppressed, and *Beta^v^* reflects the per partnership transmission from partners who are virally suppressed.

Partnership type is shown in the Supplementary Table S1.The yearly partner exchange rate per individual in gender class *i* and sexual activity class *j* for each partnership type π is given by $C_{ij\pi}$.

The mixing elements ($\rho_{ii^{'}jj^{'}\pi}$) specify the patterns of mixing between sexual activity classes by partnership type (π), which is schematically depicted in Figure 1a of the main text.

Casual (π=3) and main (π=4) partnerships are assigned (and balanced) using a mixing matrix based on Garnett *et al* [1]. A fraction of clients form casual partnerships with individuals in the high-frequency and intermediate-frequency MP class (π=3). Individuals in the high-frequency MP class form casual partnerships with clients and members of the opposite sex in the high-frequency and intermediate-frequency MP class (π=3). Individuals in the intermediate-frequency MP class form casual partnerships with clients and members of the opposite sex in the high-frequency and intermediate-frequency MP class (π=3).The mixing elements for these fixed partnerships are shown in Table S1.

The probability of forming a casual or main partnership with a given activity class of the opposite sex is given by:

$\rho_{ii^{'}jj^{'}\pi}={prob}_{ij}^{\pi}\left( \vartheta\varphi+(1-\varphi)\left( \frac{C_{i^{'}j^{'}\pi=1}N_{i'j'}{pm}_{i'j'}}{\sum_{i^{'}=1}^{2} \sum_{j^{'}=1}^{l} C_{i^{'}j^{'}\pi=1}N_{i'j'}{pm}_{i'j'}} \right) \right); \pi\in\{3,4\}$ (22)

Where, ${prob}_{ij}^{\pi}$ = the fraction of individuals in each activity class who will form a casual (π=3) or a main (π=4) partnership. $\vartheta$=1 if j=j’ and $\vartheta$=0 if j≠j’. $\varphi=0$ (proportionate mixing). The partnerships are constrained such that partnerships offered by males must equal the total number of partnerships offered by females, by partnership type:

And,

$\sum_{\pi=1}^{4} \sum_{i^{'}i}^{2} \sum_{j^{'}=1}^{l} \rho_{ii^{'}jj^{'}\pi}=1$ ; i∈{1,2}*,* j∈{1,2,3,*l*} (23)

Only FSWs and clients formed regular (π=1) and occasional (π=2) commercial partnerships. The frequency of occasional commercial partners/year among FSWs (C_211_), and the ratio of regular commercial partners for every occasional commercial partner among clients (*ratio_ro_com*) are pre-set. Then:

$$C_{111}=\frac{N_{21}{{prob}_{21}^{1}C}_{211}}{{{prob}_{11}^{1}N}_{11}}$$

$$C_{112}=C_{111}ratio\_ro\_com$$

$$C_{212}=\left( \frac{N_{11}}{N_{21}} \right)\left( \frac{{prob}_{11}^{2}}{{prob}_{21}^{2}} \right)C_{112}$$

Note that *ratio_ro_com* is varied during model calibration to provide a calibrated value of the C_112_, C_212_ (main text Table 1).

The per-partnership transmission probability of HIV transmission is given by the following:

${Beta}_{ii^{'}jj^{'}\pi}^{z,q}=\kappa_{\pi}\left( \left[ 1-\beta_{ii^{'}}^{z,u, condom} \right]^{\alpha_{\pi}} \right)+ \left( 1-\kappa_{\pi} \right) \left\{ {ps}_{ij}{ps}_{i^{'}j^{'}}\left( \left[ 1-{rr}^{s}{rr}^{i}\beta_{ii^{'}}^{z,q} \right]^{\alpha_{\pi}} \right)+ {ps}_{ij}\left( 1-{ps}_{i^{;}j^{'}} \right)\left( \left[ 1-{rr}^{s}\beta_{ii^{'}}^{z,q} \right]^{\alpha_{\pi}} \right)+\left( 1-{ps}_{ij} \right){ps}_{i^{;}j^{'}}\left( \left[ 1-{rr}^{i}\beta_{ii^{'}}^{z,q} \right]^{\alpha_{\pi}} \right)+ \left( 1-{ps}_{ij} \right)\left( 1-{ps}_{i^{;}j^{'}} \right)\left( \left[ 1-\beta_{ii^{'}}^{z,q} \right]^{\alpha_{\pi}} \right) \right\} ;q \in\{u,v\}$ (24)

$\beta_{ii^{'}}^{z,q, condom}=\left( 1-eff\_condom \right)\beta_{ii^{'}}^{z,q}; q \in\{u,v\}$ (25)

$\beta_{ii^{'}}^{z,v}=\left( 1-eff\_art \right)\beta_{ii^{'}}^{z,u}$ (26)

Where$\beta_{ii^{'}}^{z,q}$ is the transmission probability per unprotected sex-act to a susceptible individual in sex *i* from an individual of the opposite sex *i’* who is infectious and in stage z , and either virally suppressed (*q=v*) or not virally suppressed (*q=u*). α_π_ represents the number of sex acts within each type of partnership. The prevalence of a sexually transmitted infection (specifically, HSV-2) is given by *ps_ij_*, which confers an increase in HIV susceptibility (*rr^s^*) and/or an increase in HIV infectiousness (*rr^i^*) per sex-act.

1.3 Developing plausible synthetic epidemics using the 4-stage dynamical model

The 4-stage dynamical model was simulated assuming a linear increase in cART initiation (from ART roll-out to coverage achieved by 2011 in each setting), and a linear increase in condom-use from 1995 to levels achieved by 2008 (Belgaum), and 2005 (Lesotho, Kisumu), as per Table 1 in the main text.

To ensure each synthetic epidemic reflected a plausible scenarios, the model was calibrated by minimizing least squares difference between the empirical data (trends in HIV prevalence by risk-groups and overall ART coverage) and the 4-stage DM model outputs for each simulated setting. The model calibration was also restricted to the weighted average for observed partner change rate in the total MP group (intermediate- and high-frequency). For each setting, the model was calibrated to obtain parameter inputs which were unavailable or were least reliable (Table 1 in main text).

Following calibration, HIV transmission within the following types of partnerships were “turned off” (${Beta}_{ii^{'}jj^{'}\pi}^{z,u}$ = 0 ) at the start of each epidemic.

1. π=1 and π=2 [i.e. the absence of commercial sex]
2. π=1 and π=2 and π=3 [i.e. absence of commercial and casual sex]
3. π=3 [i.e. absence of casual sex]

The synthetic epidemics were then classified as “concentrated”, “mixed”, and “generalized”, as defined in the main text (Methods). The modeled epidemic curves are shown in Supplementary Figure S2, Figure S3, Figure S4, and Figure S5.

1.4 Modifying the 4-stage dynamical to the 1-stage dynamical model

The 1-stage dynamical model uses the same parameters as the 4-stage dynamical model (i.e. the synthetic epidemic), with the following exception: in the 1-stage dynamical model, a uniform per sex-act transmission probability is used (calculated as a weighted average across the 4 stages of HIV).

$\beta_{ii^{'}}^{average,u}=\frac{\sum_{z=1}^{4} \beta_{ii^{'}}^{z,u}\frac{1}{{(\gamma}^{z}+\phi^{z})}}{\sum_{z=1}^{4} \frac{1}{{(\gamma}^{z}+\phi^{z})}}$ (27)

1.5 Model analysis (Population Attributable Fraction) using the 4-stage dynamical model

To measure the population attributable fraction (PAF) of specific subgroups to overall transmission, the cumulative number of infections (CI) in the total population is calculated when${Beta}_{ii^{'}jj^{'}\pi}^{z,u}$ is given by equation 24 (full model), and comparing the CI when${Beta}_{ii^{'}jj^{'}\pi}^{z,u}$ = 0 from *time= mot* for each subgroup given by *ij*. The PAF is calculated as:

${CI}_{full}=\int_{mot}^{t} \sum_{i=1}^{2} {\sum_{j=1}^{l} S_{ij}\lambda}_{ij}dt$ *i*≠*i’* and j∈{1,2,3,*l*} (28)

${CI}_{no transmission from ij}=\int_{mot}^{t} \sum_{i=1}^{2} {\sum_{j=1}^{l} S_{ij}\lambda}_{ij}dt$ and ${\beta eta}_{ii^{'}jj^{'}\pi}^{z,u}=0$ for a given *ij* (29)

${PAF}_{t}^{from ij}= 100(\frac{{CI}_{full-}{CI}_{no transmission from ij}}{{CI}_{full}}$) (30)

1.6 Model analysis (Benchmark Modes of Transmission [MOT] metric)

The benchmark MOT metric was obtained from the synthetic data generated by the 4-stage dynamical model. The number of HIV infections acquired within each subgroup over 1 year measured from the year of the MOT (*time=mot*) is given by the following:

${DM\_I}_{ij}=\sum_{=1}^{4} \sum_{j'=1}^{l} \int_{mot}^{mot+1} \rho_{ii^{;}jj^{;}\pi}\sum_{z=1}^{4} \left\{ 1-\left[ \left( \frac{{Beta}_{ii^{'}jj^{'}\pi}^{z,u}{Iu}_{i^{'}j^{'}}^{x}+ {Beta}_{ii^{'}jj^{'}\pi}^{z,v}{Iv}_{i^{'}j^{'}}^{x}}{N_{i^{'}j^{'}}} \right)+\left( 1-\frac{\left( {Iu}_{i^{'}j^{'}}^{x}+{Iv}_{i^{'}j^{'}}^{x} \right)}{N_{i^{'}j^{'}}} \right) \right]^{C_{ij\pi}} \right\}\left( S_{\mathrm{ij}} \right)dt ; i i’ \mathrm{and}j \{1,2,3,l\}$ (31)

Where *mot* = current year, and 1 year is the time step over which we want to ascertain how many new infections will occur in activity *j* as a result of a partnership with individuals in activity class *j’*. If $I_{i^{'}j^{'}}^{z}$ and S_ij_ do not change very much over the 1 year time step, then equation 31 approximates to:

${DM\_I}_{ij}=\sum_{=1}^{4} \sum_{j'=1}^{l} \rho_{ii^{;}jj^{;}\pi}\sum_{z=1}^{4} \left\{ 1-\left[ \left( \frac{{Beta}_{ii^{'}jj^{'}\pi}^{z,u}{Iu}_{i^{'}j^{'}}^{x}+ {Beta}_{ii^{'}jj^{'}\pi}^{z,v}{Iv}_{i^{'}j^{'}}^{x}}{N_{i^{'}j^{'}}} \right)+\left( 1-\frac{\left( {Iu}_{i^{'}j^{'}}^{x}+{Iv}_{i^{'}j^{'}}^{x} \right)}{N_{i^{'}j^{'}}} \right) \right]^{C_{ij\pi}} \right\}\left( S_{\mathrm{ij}}(time=mot) \right); i i’ \mathrm{and}j \{1,2,3,l\}$ (32)

1.7. Model analysis (1-stage dynamical model MOT metric)

The 1-stage dynamical model MOT metric was obtained in the same way as described in section 1.6, using the 1-stage dynamical model.

**2. MOT models**

We developed two MOT models: a complex MOT (cMOT) in keeping with the population risk stratification and partnerships defined in the dynamical model (main text Figure 1b), and a generic MOT (gMOT, main text Figure 1c) in keeping with the template provided by UNAIDS [2]. The MOT models were constructed and programmed in Stata 11 (StataCorp).

2.1 Complex MOT model (cMOT)

For the one-year period of examination starting at *time*=*mot*, individuals in each gender and sexual activity class are divided into *n*^ij^ subsections as depicted in the main text Figure 1b. In the cMOT, individuals are allowed to have ≥1 type of HIV exposure in the year the MOT metric is measured.

$S_{ij}=\sum_{n=1}^{n^{ij}} S_{ijn}$ (33)

$I_{ij}=\sum_{n=1}^{n^{ij}} I_{ijn}$ (34)

As described by Gouws *et al* [2,3], the number of HIV infections within acquired by each risk group is given by the following:

$${cMOT_{I}}_{ij}=\sum_{n=1}^{n^{ij}} \sum_{\pi=1}^{\pi} \sum_{j^{'}=1}^{l} \sum_{q=u}^{v} \begin{aligned} \rho_{ii^{;}jj^{;}\pi}S_{ijn}(time=mot) \{1-[ \left( \frac{{\sum_{z=1}^{4} I}_{i^{'}j^{'}}^{z,q}\left( time=mot \right)}{N_{i^{'}j^{'}}} \right)( \\ \left( 1-\kappa_{\pi} \right) [ ( {ps}_{i^{'}j^{'}} p_{ij} \left( 1-{{rr}^{i}{rr}^{s}\beta}_{ii^{'}j}^{average,q} \right)^{\alpha_{\pi}}) \end{aligned}$$

$$+ ( {(1-ps}_{i^{'}j^{'}}) p_{ij} \left( 1-{{rr}^{s}\beta}_{ii^{'}j}^{average,q} \right)^{\alpha_{\pi}})$$

$$+ \left( {ps}_{i^{'}j^{'}}\left( 1- p_{ij} \right)\left( 1-{{rr}^{i}\beta}_{ii^{'}j}^{average,q} \right)^{\alpha_{\pi}} \right)$$

$$+ ( {(1-ps}_{i^{'}j^{'}})(1- p_{ij}) \left( 1-\beta_{ii^{'}j}^{average,q} \right)^{\alpha_{\pi}}) ]$$

+$\kappa_{\pi} [{(1-\beta_{ii^{'}j}^{average,q, condom})}^{\alpha_{\pi}}]$ +

${(1-(\frac{{\sum_{z=1}^{4} I}_{i^{'}j^{'}}^{z,q}\left( time=mot \right)}{N_{i^{'}j^{'}}}))]}^{C_{ij\pi}}\}$

(35)

Where *n^ij^* are the divisions of the *i* activity class (main text Figure 1b-c), such that each combination of *i,j,n* can only have one type of partnership (π). In order to directly compare the cMOT metric to the benchmark, the values of the input parameters for the cMOT model were obtained from the simulated outputs of the 4-stage dynamical model (i.e. the synthetic epidemic), at each specific time point in the epidemic (epidemic phase).

It can be seen under assumption of the same parameter values, the same sexual structure (cMOT), and uniform HIV infectivity (1-stage DM compared with the cMOT), that equation 32 approximates equation 35 when:

1. ΔS_ijn_ ~ 0 [where ΔS_ijn_ = S_ijn_(*time=mot*) - S_ijn_(*time=mot+1*)], and $\Delta{Iu}_{i^{'}j^{'}}^{z} 0$ [where ${\Delta I}_{i^{'}j^{'}}^{z}$= $I_{i^{'}j^{'}}^{z}$ (*time=mot*) - $I_{i^{'}j^{'}}^{z}$ (*time=mot+1*)]

Mismatch in the estimates of the fraction of new infections acquired within a subgroup (MOT metric) from the benchmark MOT metric (from the dynamical model) and the cMOT metric would be most evident when the above assumptions do not hold: for example, when S_ijn_ changes due to acquired infections (high per-capita risk of HIV transmission) and due to rapid turn-over in the subgroup, as in the case of FSWs.

2.1 Generic MOT model (gMOT)

In the gMOT, risk-groups are allowed only one type of HIV exposure in the year the MOT is measured. Individuals in each gender and sexual activity class are divided into *n*^ij^ subsections as depicted in the main text Figure 1c. Therefore, the following simplifications to the cMOT are made in keeping with the generic template [2].

- The gMOT takes into account co-infection with a sexually transmitted infection, co-infection only increases HIV infectiousness.
- Condom-use is incorporated such that condom coverage, $\kappa_{\pi}$, reflects the fraction of sex-acts protected within each partnership.
- Clients can only become infected from FSWs via one type of commercial partnership
- FSWs can only become infected from clients via one type of commercial partnership
- The two multiple partnership classes are collapsed into one class, and can only become infected via casual partnerships.

Therefore, commercial partnerships were modified as follows: π=1 and π=2 were amalgamated into π=commercial. The number of sex acts per partnership per year in this amalgamated commercial partnership was set to equal 1 (= to # of sex acts in an occasional commercial partnership).

$C_{21commercial}=C_{21\pi=1}+ C_{21\pi=2}$ (36)

$C_{11commercial}=\frac{\left( C_{21\pi=1}+ C_{21\pi=2} \right)N_{21}(time=mot)}{N_{11}(time=mot)}$ (37)

In the gMOT, Casual partnerships only form between members of the MP class, and thus modified as follows:

$N_{i}^{mp}=N_{i2}+N_{i2}, at time=mot$ (38)

$I_{i}^{z,mp}=\sum_{z=1}^{4} I_{i2}^{z}+I_{i3}^{z}, at time=mot$ (39)

$S_{i}^{mp}=S_{i2}+S_{i2}, at time=mot$ (40)

$C_{2casual}^{mp}=C_{223}\left( \frac{N_{22}}{N_{22}+N_{23}} \right)+ C_{233}\left( \frac{N_{23}}{N_{22}+ N_{23}} \right)$ (41)

$C_{1casual}^{mp}= \frac{\left( C_{2casual}^{mp} \right)N_{2}^{mp} (time=mot)}{N_{1}^{mp}(time=mot)}$ (42)

Therefore, equation 35 is modified for the gMOT:

${gMOT_{I}}_{ij}=\sum_{n=1}^{n^{ij}} \sum_{\pi=1}^{4} \sum_{j^{'}=1}^{l} S_{ijn}\left( time=mot \right)= \left\{ 1-\left\lceil\begin{aligned} \left( \frac{{\sum_{z=1}^{4} I}_{i^{'}j^{'}}^{u}\left( time=mot \right)}{N_{i^{'}j^{'}}} \right)\left( \begin{aligned} \left( {ps}_{i^{'}j^{'}} \left( 1-{{rr}^{i}\beta}_{ii^{'}j}^{average,u} \right)^{\alpha_{\pi}\left( 1-\kappa_{\pi} \right)} \right) \\ +\left( {(1-ps}_{i^{'}j^{'}} ) \left( 1-\beta_{ii^{'}j}^{average,u} \right)^{\alpha_{\pi}\left( 1-\kappa_{\pi} \right)} \right) \end{aligned} \right) \\ +\left( \frac{{\sum_{z=1}^{4} I}_{i^{'}j^{'}}^{v}\left( time=mot \right)}{N_{i^{'}j^{'}}} \right)\left( \left( 1-\beta_{ii^{'}j}^{average,v} \right)^{\alpha_{\pi}\left( 1-\kappa_{\pi} \right)} \right) \\ + \left( 1-\left( \frac{{\sum_{z=1}^{4} I}_{i^{'}j^{'}}^{z}\left( time=mot \right)}{N_{i^{'}j^{'}}} \right) \right) \end{aligned} \right\rceil^{C_{ij\pi}} \right\}$ (43)

**3. Intervention policies and implementation**

HIV prevention policies were chosen on the basis of the generic MOT results (MOT metric from the gMOT) and are described in the main text (Methods). Finite resources were applied to compare intervention impact from a policy change in 2012. For illustrative purposes, we applied a generic intervention that reduces HIV transmission per sex-act by 80%. If the policy prioritized the low-activity population (policy 1, policy 2), then the generic intervention was restricted to the currently low-activity class. If the policy prioritized the known epidemic drivers (policy 3), the generic intervention was applied within the respective partnerships (commercial, casual, or both commercial and casual) to the relevant subgroups that represent the epidemic drivers (FSWs-clients; individuals within multiple partnerships).

**References**

1. Garnett GP, Anderson RM (1994) Balancing sexual partnerships in an age and activity stratified model of HIV transmission in heterosexual populations. IMA J Math Appl Med Biol 11: 161-192

2. UNAIDS (2012) Modelling the expected short-term distribution of incidence of HIV infections by exposure group. Geneva. Available: http://www.unaids.org/en/dataanalysis/datatools/incidencebymodesoftransmission/ Accessed 1 December 2013

3. Gouws E, White PJ, Stover J, Brown T (2006) Short term estimates of adult HIV incidence by mode of transmission: Kenya and Thailand as examples. Sex Transm Infect 82: iii51-iii55. doi: 10.1136/sti.2006.021064
